# Systems-Based Approaches to Unravel Networks and Individual Elements Involved in Apple Superficial Scald

**DOI:** 10.3389/fpls.2020.00008

**Published:** 2020-02-13

**Authors:** Evangelos Karagiannis, Georgia Tanou, Federico Scossa, Martina Samiotaki, Michail Michailidis, Maria Manioudaki, François Laurens, Dominique Job, Alisdair R. Fernie, Mathilde Orsel, Athanassios Molassiotis

**Affiliations:** ^1^Laboratory of Pomology, Department of Agriculture, Aristotle University of Thessaloniki, Thessaloniki, Greece; ^2^Institute of Soil and Water Resources, ELGO-DEMETER, Thessaloniki, Greece; ^3^Department Willmitzer, Max-Planck-Institute of Molecular Plant Physiology, Potsdam-Golm, Germany; ^4^Council for Agricultural Research and Economics, Research Center for Genomics and Bioinformatics, Rome, Italy; ^5^Institute for Bioinnovation, Biomedical Sciences Research Center “Alexander Fleming”, Vari, Greece; ^6^Institut de Recherche en Horticulture et Semences (IRHS), UMR 1345, INRA, Agrocampus-Ouest, Université d’Angers, Beaucouzé, France; ^7^Centre National de la Recherche Scientifique – Université Claude Bernard Lyon 1 – Institut National des Sciences Appliquées-Bayer CropScience, Lyon, France

**Keywords:** apple fruit, ethylene inhibition, glutathione *S*-transferases, metabolites, ozone, proteomics, ripening, superficial scald

## Abstract

Superficial scald is a major physiological disorder in apple fruit that is induced by cold storage and is mainly expressed as brown necrotic patches on peel tissue. However, a global view of the gene-protein-metabolite interactome underlying scald prevention/sensitivity is currently missing. Herein, we have found for the first time that cold storage in an atmosphere enriched with ozone (O_3_) induced scald symptoms in ‘Granny Smith’ apple fruits during subsequent ripening at room temperature. In contrast, treatment with the ethylene perception inhibitor 1-methylcyclopropene (1-MCP) reversed this O_3_-induced scald effect. Amino acids, including branched-chain amino acids, were the most strongly induced metabolites in peel tissue of 1-MCP treated fruits. Proteins involved in oxidative stress and protein trafficking were differentially accumulated prior to and during scald development. Genes involved in photosynthesis, flavonoid biosynthesis and ethylene signaling displayed significant alterations in response to 1-MCP and O_3_. Analysis of regulatory module networks identified putative transcription factors (TFs) that could be involved in scald. Subsequently, a transcriptional network of the genes-proteins-metabolites and the connected TFs was constructed. This approach enabled identification of several genes coregulated by TFs, notably encoding glutathione *S*-transferase (GST) protein(s) with distinct signatures following 1-MCP and O_3_ treatments. Overall, this study is an important contribution to future functional studies and breeding programs for this fruit, aiding to the development of improved apple cultivars to superficial scald.

## Introduction

Apple (*Malus* × *domestica* Borkh.) is one of the most marketable and popular fruit crops; over 80 Mt are produced per year throughout the temperate regions of the world (FAO statistic, http://faostat3.fao.org). Cold storage of susceptible apple cultivars is widely used to delay apple fruit ripening and senescence programs (Zermiani et al., 2015). However, the long-term cold storage of apples leads to development of superficial scald, a major physiological disorder that is characterized by necrosis of the hypodermal cortical tissue. These symptoms could be developed during cold storage and become more evident during subsequent ripening at room temperature, thus reducing the market quality of susceptible apple cultivars, such as ‘Granny Smith’ (Lurie and Watkins, 2012).

The complex regulation of scald is one of the most important topics in fruit research, but this phenomenon is still unclear (Storch et al., 2017). Early studies linked the oxidation of *α*-farnesene, a natural volatile compound present in the wax of the fruit, and its oxidation into conjugated trienols (CTols) and ketone 6-methyl-5-hepten-2-one (MHO) during scald development (Rowan et al., 1995). Currently, this *α*-farnesene theory has been debated, and it has been suggested that several other pathways could directly or indirectly be involved in scald disorder. It has been recently proposed that cold-induced oxidative stress in the peel of ‘Granny Smith’ are linked to the accumulation of chlorogenic acid in the vacuole, which in turn could react with polyphenol oxidase (PPO), leading to the peel browning that is typical of scald (Busatto et al., 2018). Further, inhibition of ethylene perception by 1-MCP has been shown to stimulate the production of antioxidant compounds to scavenge reactive oxygen species (ROS), the synthesis of fatty acids to stabilize plastid and vacuole membranes against cold, and the accumulation of sorbitol that can act as a cryoprotectant (Busatto et al., 2018).

High-throughput biological approaches like transcriptomics, proteomics, and metabolomics have been widely used to explore low temperature storage-responsive mechanisms in fruits (Molassiotis et al., 2013). Analyses from omics perspectives of apple scald have been conducted, including RNAseq (Busatto et al., 2018), proteomics (Du et al., 2017) and metabolomics (Rudell et al., 2009; Busatto et al., 2014; Gapper et al., 2017). Despite such progress, combination analyses of these different omics approaches and of the corresponding data sets by network construction and characterization of regulatory pathways in relation to scald have not yet been reported.

In the present work, we aimed to characterize scald responses in apple fruit, to uncover the metabolic relationships between scald-affected and healthy phenotypes, and thus we applied to ‘Granny Smith’ apples individual and combined treatments with the *1*-*methylcyclopropene* (*1*-*MCP*), an *inhibitor* of *ethylene perception*, as well as with ozone (O_3_), which is a powerful oxidant compound known to inhibit ethylene biosynthesis in kiwifruit (Minas et al., 2014; Minas et al., 2018). We revealed, for the first time, that ozone induced scald symptoms while 1-MCP totally reversed this O_3_-stimulated scald effect. By using this novel experimental system and by employing a multiomics approach and regulatory module networks analysis we identified pathways and networks as well individual elements (putative transcription factors/genes/proteins/metabolites) involved in scald prevention/sensitivity.

## Materials and Methods

### Experimental Design and Treatments

**Figure 1:**
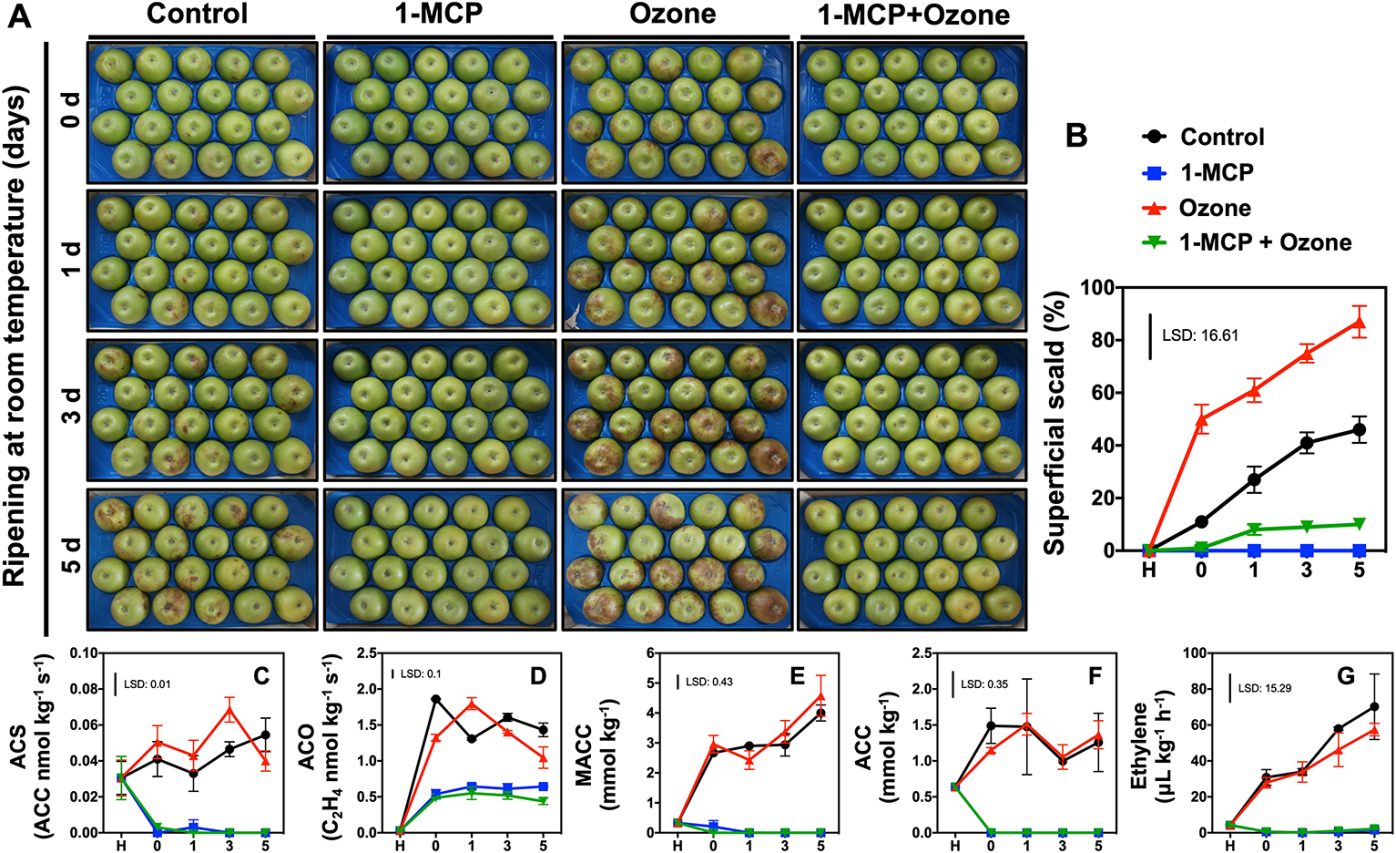
Regulation of scald development and ethylene metabolism in response to chemical treatments. Apples (cv. Granny Smith) were treated or not with 1-MCP (1 μl L^-1^) and cold stored (0°C) for 6 months in the presence or absence of O_3_ atmosphere (0.3 μl L^-1^). Subsequently the fruits were transferred at room temperature to characterize their ripening and scald development for up to 5 days. Phenotypes of apple fruits picturing different expression of scald syndrome **(A)**, percentage of apple’s peel affected by superficial scald **(B)**, changes in enzymatic activities of ACC synthase (ACS) **(C)** and ACC oxidase (ACO) **(D)** as well as steady-state level of 1-malonyl-aminocyclo-propane-1-carboxylic acid (MACC) **(E)** and 1-aminocyclopropane-1-carboxylic acid ACC **(F)**, and ethylene concentration **(G)**. The vertical bar represents the least significant difference (LSD, P = 0.05) of three independent biological replications, which was used for means comparison between the different treatments and time points, while the vertical bars at each time point represent the standard error of the means (SEM).

*‘Granny Smith’ apple* fruits were harvested at physiologically mature stage (firmness: 7.59 kg ± 0.12 kg; soluble solids content: 11.8% ± 0.1%, titratable acidity (malic acid, %): 0.77% ± 0.02%; dry weight: 14.03% ± 0.02%) from a commercial orchard at Imathia region (North Greece). Fruits were randomly divided into two groups of 250 fruits each. Apples of the first group were treated with 1-MCP (1 μl L^-1^, SmartFresh, AgroFresh Inc., Rohm and Haas, Spring House, USA) for 24 h at 0°C, according to manufacturer’s instructions, whereas the second group remained untreated (control). Fruits from each group were cold stored (0°C, 95% RH) in two separated cold rooms in the absence or presence of enriched O_3_ atmosphere (0.3 μl L^-1^) through a dedicated system of continuous O_3_ generation and monitoring (ozone generator model COM-AD-04 and ozone analyser model MP-6060, Anseros Klaus Nonnenmacher GmbH, Tübingen, Germany) for 6 months. Following cold storage, fruits from the four different treatments (control, 1-MCP, O_3_, 1-MCP+O_3_) were analyzed for the development of scald symptoms after 0, 1, 3, and 5 days (d) ripening at room temperature (20°C). Each analysis at room temperature immediately after the cold storage (0 d at room temperature) compared to harvest time was named “cold storage effect.” Sampling at postcold ripening period and before scald visual appearance (0 d at room temperature) was defined as “presymptomatic period,” while sampling after 5 d ripening in which all scald-affected fruits (**Figure 1A**) was defined as “symptomatic period.” For each treatment, three replicates of the whole peel tissue per fruit were isolated from seven apples, and subsequently frozen in liquid nitrogen and stored at -80°C for further analysis.

### Physiological and Statistical Analyses of Apple Ripening

Fruit firmness, soluble solids concentration (SSC), titratable acidity (TA, malic acid %) and respiration rate were measured according to Karagiannis et al. (2018a). Statistical analysis was conducted using SPSS 20.0 (SPSS, Chicago, IL, USA). The means of three independent biological replications of seven fruits per replication were used for firmness, SSC and TA analysis, while three independent biological replications of two fruits per replication were used for respiration rate and ethylene determination. Subsequently, data were subjected to analysis of variance and least significant differences (LSD with SEM) at 5% level were used for means comparison.

### Evaluation of Superficial Scald Symptoms

The percent of scald surface was recorded as incidence and severity of affected fruits using a scale where 0 = none, 1 = 1%–10%, 2 = 11%–33%, 3 = 34%–66%, and 4 = 67%–100% of the surface area (Karagiannis et al., 2018b). Statistical analysis was performed as described above (three independent biological replications of thirty fruits per replication).

### Analysis of Basic Metabolites and Enzymes of Ethylene Biosynthesis (Yang Cycle)

Ethylene production was measured using a gas chromatograph system (GC-2014ATF Shimadzu) (Tanou et al., 2017). Enzymes and metabolites of the ethylene biosynthesis cycle were determined at grinded tissue and extracted as previously described by Bulens et al. (2011). Statistical analysis was performed as described above (three independent biological replications of seven fruits per replication).

### Metabolic Profiling by GC-ToF-MS Analysis

**Figure 2:**
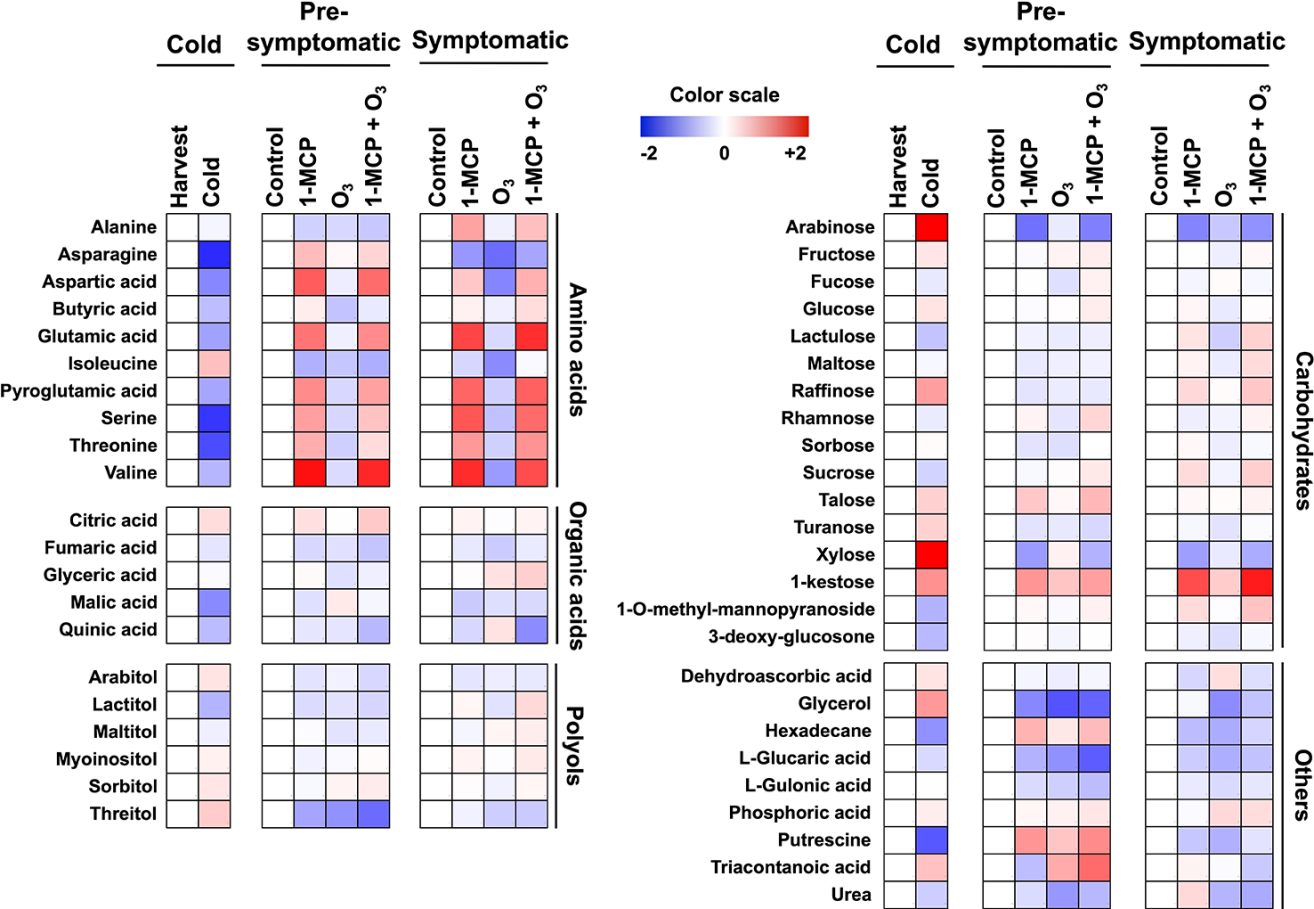
Modulation of apple peel metabolites following 1-MCP and O_3_ treatments. Changes in primary metabolites in peel tissue exposed to 1-MCP and O_3_ at “cold”, “presymptomatic”, and “symptomatic” period. Treatments and sampling were performed as described in **Figure 1** and under “Materials and Methods.” A color scale that is proportional to the log2 ratio of each identified metabolite shows the fold change between treated and control fruits. Mean values of three independent measurements for each treatment were analyzed between the treatments and the control. Relative values for each metabolite mean are provided in **Supplementary Table 1**.

Primary metabolites, collected at commercial harvest point as well as at 0 and 5 d of ripening, were extracted and derivatized from 250 mg of freeze dried peel tissue and analyzed using a gas chromatography time of flight-mass spectrometry (GC-ToF-MS) according to established procedures (Lombardo et al., 2011). Peaks were identified using Tagfinder (Luedemann et al., 2008) and their identity confirmed using the mass spectral tags in the MPI-MP Golm database (Hummel et al., 2010). Metabolite relative amounts were normalized on the peak area of the internal standard ribitol according to the procedure outlined in Roessner et al. (2000). The normalized values of the 46 primary metabolites were also analyzed by one-way ANOVA followed by Duncan’s test to detect significant differences (P < 0.05). Further details regarding the protocols for metabolic profiling and the full data set of identified metabolites are reported in **Supplementary Table 1** following established guidelines (Fernie et al., 2011). Mean values of five independent samples for each stage were expressed as the log2-transformed ratio between each treatment compared to control at different stage (**Figure 2**).

### Quantitative Proteome Analysis

At the same time points sampled for metabolomic analysis, peel tissue (5 g) were used for protein extraction based on phenol extraction protocol at total volume of 12 ml (Karagiannis et al., 2016). Two-dimensional electrophoresis was performed according to Tanou et al. (2010) using a BIO-RAD system. Following silver nitrate staining (Minas et al., 2016), 2DE-gels were scanned with Bio-Rad GS-800 Calibrated Densitometer equipped with PDQuest Advanced 2-DE Gel Analysis Software (**Supplementary Figure 2**). Statistical analysis of three independent biological replications was done by one-way analysis of variance significance (P ≤ 0.05) and individual means were compared using Student’s t-test (P ≤ 0.05). In addition to the p value criterion, spots showing an at least 2.5 fold change of volume were considered as statistically significant (Tanou et al., 2015).

Spots of interest underwent tryptic in-gel digestion and peptide fragments analyzed by MALDI-MS in a TOF-MS (Ultraflex II, Bruker Daltonics, Bremen, Germany) as detailed by Tanou et al. (2017). Peptide mixtures were analyzed in a MALDI-TOF mass spectrometer (Autoflex-Speed, Bruker Daltonics). Raw files were searched against the Uniprot *Malus domestica* protein database using the (MASCOT Server v2.0). The mass error tolerance on the Mascot server was set to 25 μl L^-1^ methionine oxidation was considered as a variable modification and cysteine carbamidomethylation was considered as a fixed modification. All peptide sequences, accession numbers, Mascot scores and sequence coverage are provided in **Supplementary Table 4**. For nonidentified spots, peptide fragments were analyzed by LC-MS/MS using a LTQ Orbitrap XL Mass spectrometer (Thermo Fisher Scientific, Bremen, Germany) coupled online with a nanoLC Ultimate 3000 chromatography system (Dionex, Sunnyvale, CA) (Ainalidou et al., 2016). Raw files were searched against the ncbi *Malus domestica* protein database using PD 1.4 and the SEQUEST HT search engine. Protein identification required minimal XCorr values of 2.0, and 2.5 for charge states of doubly, and triply precursor ions respectively (**Supplementary Table 4**). Where protein annotations were missing, manual protein BLAST was performed against the current databases. Identifications are based on at least two peptides per protein. When presented, identifications based on single peptide additional information are provided (**Supplementary Table 5**). This work is MIAPE compliant. The mass spectrometry proteomics data have been deposited to the ProteomeXchange Consortium *via* the PRIDE [1] partner repository with the dataset identifier PXD016849.

### RNA Extraction, Amplification, and Hybridization

Total RNA was extracted from 1 g of grinded frozen fruit peel tissue plus 5 ml of extraction buffer, as described (Nobile et al., 2011; Segonne et al., 2014). Total mRNAs were amplified, labelled and cohybridized according to Celton et al. (2014) as follows: amplified aRNAs were produced with Message AmpII aRNA amplification kit (Ambion) from 200 ng of total RNA. Then, each aRNA (5 μg) was retrotranscribed and labelled with either Cyanine-3 or Cyanine-5 fluorescent dye (Interchim, Montluçon, France). Labelled samples were combined as 30 pmol for each dye and cohybridized to the Agilent microarray AryANE v2.0 (GPL26715) containing 135,000 60-mers oligonucleotide probes as described (Daccord et al., 2017). The experimental design included four comparisons with respectively 1-MCP, O_3_, 1-MCP+O_3_ treated fruits versus control fruits and 1-MCP+O_3_ treated fruits versus O_3_ treated fruits, all at presymptomatic/prosymptomatic stage after 6 months of cold storage. Each comparisons included two sampling repetitions using the dye switch statistical technique according to (Mary-Huard et al., 2008).

### Microarray Analysis

The Agilent Feature Extraction 11.5 software was used to extract data files from from the scanned images obtained using the MS200 microarray scanner (Roche Nimblegen). All statistical analyses were conducted based on a dye switch approach as described (Daccord et al., 2017) with the R software (R development Core Team, 2009). Briefly, data were normalized with the Lowess’s method, and differential expression analyses were carried out using the lmFit function and the Bayes moderated t test using the R package LIMMA (Smyth, 2005) from the Bioconductor project. Genes were considered differentially expressed if the t-test *P-*values of the paired sample were below 1%. Genesis software was used to visualize results. Functional classification was based on Mapman ontology (Usadel et al., 2005; Zhao et al., 2019). Microarray data have been submitted to the Gene Expression Omnibus under the accession number GPL26715. Both antisense (AS) and sense (S) probes were designed for each sample, and 16.5% of the significantly expressed probes corresponded to antisense (AS) transcripts (**Supplementary Table 5**). Previous studies (Dheilly et al., 2016) demonstrated that AS transcripts were likely to be involved in small interfering RNA (siRNA), dependent on the negative regulation of the coding mRNAs. Subsequently, this current study considers only the genes with sense (S) probes as differentially expressed transcripts.

### Gene Expression Quantification by RT-qPCR

The RNA samples used for the microarrays experiments were treated with 2 U of DNAse I (Promega, USA) and cDNAs were synthesized from 1 μg of DNA-free-RNA with oligo(dT) 15 and 200 U of MMLV-RT (Promega) according to Segonne et al. (2014). For each cDNA, qPCR experiments were carried out according to Segonne et al. (2014) and Dheilly et al. (2016). Based on microarray results, seven genes of interest (GOI) were selected for being differentially expressed in two or more comparisons, while five genes were selected for never being differentialy expressed in all four comparisons to be used as reference genes (Celton et al., 2014; Vergne et al., 2014; Dheilly et al., 2016). Primer pairs for selected genes (**Supplementary Table 6**) were tested for their respective specificity and efficiency using a dilution curve method. Only primer pairs with efficiencies higher than 85% were retained for further analysis. Relative expression levels of three independent biological replicates were calculated according to Livak and Schmittgen (2001).

### Network Analysis

Transcriptional regulatory networks including modules and regulatory interactions were inferred among transcripts, proteins and metabolites, using the probabilistic module networks frameworks LeMoNe that uses ensemble based probabilistic optimization techniques to identify clusters of coexpressed transcripts as well as their regulators (Espley et al., 2007). Data set used for the network analysis were the log2 ratio from the list of the differentially regulated genes selected by the 1-MCP, O_3_ and 1-MCP+O_3_ comparisons to treatment, as well as the proteins and the metabolites listed in **Supplemental Tables 1**, **3**, and **5**, respectively. Annotations for transcription factors were downloaded from the Transcription Factor Database (http://planttfdb.cbi.pku.edu.cn).

## Results

### Superficial Scald Symptoms Development

Phenotypic (**Figure 1A**; the same fruits are presented at different time points) and scald evaluation (**Figure 1B**) indicated that control and especially ozone-treated fruits (without 1-MCP exposure) exhibited scald symptoms at 0 d ripening. At day 5, approximately 87% O_3_-treated apples developed scald symptoms while control fruits showed about 46.0% scald (**Figure 1B**). Nevertheless, 1-MCP-treated apples, in the presence or in the absence of O_3_, substantially reduced scald injury (10%). Scald symptoms were low (9.5%) in fruit exposed to combined 1-MCP and O_3_ application (1-MCP+O_3_ treatment) while no scald was detected in fruit treated with 1-MCP (**Figure 1B**).

### Effect of 1-MCP and O_3_ on Apple Climacteric Ripening

Treatment with 1-MCP inhibited ethylene biosynthesis (**Figures 1C–G**). In contrast, activities of ACS and ACO, and ACC and MACC levels along with ethylene production (**Figures 1C–G**) remained unaffected in O_3_-treated apples compared to control (**Supplementary Figure 1**).

### Metabolic Profiling in 1-MCP and O_3_-Treated Peel Tissue During Scald Development

To obtain information about the 1-MCP and O_3_ metabolic functions during scald development, primary metabolites were monitored at harvest, presymptomatic (0 d at room temperature) and symptomatic periods (5 d at room temperature) (**Figure 2**, **Supplementary Table 1**). Metabolic profiling in apple peel just after cold storage compared to harvest showed that the accumulation level of 23 metabolites varied, with 13 and 10 metabolites showing decreased and increased abundance, respectively (**Figure 2**). Several amino acids (e.g., valine, serine, threonine, and aspartic acid), sucrose (the main soluble sugar), malic acid (the main organic acid) as well as putrescine, hexadecane, 3-deoxy-glucosone, and 1-*O*-methyl-mannopyranoside decreased during cold storage (**Figure 2**; **Supplementary Table 1**). Meanwhile, metabolites whose abundance increased after cold period were mainly soluble sugars and soluble alcohols including arabinose, xylose, 1-kestose, raffinose, fructose (soluble sugars), sorbitol, threitol, glycerol, arabitol (soluble alcohols), as well as dehydroascorbic acid (**Figure 2**).

At the presymptomatic period 18, 10, and 16 metabolites showed altered abundance in response to 1-MCP, O_3,_ and 1-MCP+O_3_ treatments, respectively (**Figure 2**). 1-MCP treatment led to an increase in the amount of citric acid, talose, serine, and threonine (**Figure 2**). Ozone-affected metabolites included glyceric acid and urea (both decreased levels), while the combined treatment 1-MCP+O_3_ affected the accumulation of sucrose (increased) and arabitol (decreased). Additionally, six metabolites were commonly repressed by all treatments, including glycerol, isoleucine, fumaric acid, threitol, and the lactones of gulonic acid and glucaric acid (**Figure 2**).

At postsymptomatic stage, 12 and 10 metabolites were influenced by individual 1-MCP and O_3_ applications, respectively while 19 metabolites showed altered accumulation following 1-MCP+O_3_ treatment (**Figure 2**). In response to 1-MCP there was an increased level of kestose (a trisaccharide deriving from sucrose), alanine and myoinositol (**Figure 2**). Meanwhile, O_3_ reduced the level of glycerol, isoleucine and turanose (α-d-glucopyranosyl-(1→3)-α-d-fructofuranose, an analog of sucrose), while 1-MCP+O_3_ increased that of glyceric acid, mannopyranoside, raffinose, maltose, sucrose but decreased that of quinic acid. Finally, five metabolites including valine, arabitol, glucaric acid, arabinose, and xylose were commonly affected by all treatments (**Figure 2**; **Supplementary Table 1**).

### Time-Resolved Proteomics in Apple Peel Exposed to 1-MCP and O_3_ Treatments

To characterize the scald phenotypes (**Figure 1**), a comparative proteomic analysis of apple peel was performed. The volumes of 217 protein spots were modified by the treatments as inferred from Student’s t-test and further validation using a 2.5-fold threshold change. Following this approach, 87 proteins were uniquely identified (**Supplementary Table 3A**), which were sorted into 11 functional categories and 12 subcellular localizations according to Bevan et al. (1998) (**Supplementary Figure 3**; **Supplementary Table 3**; **Supplementary Table 4**). Multispot proteins, such as major allergen (15 spots), actin (11 spots), histone H4 (6 spots), and thiamine triazole synthase (4 spots) were also identified. Detailed information on the identified proteins is listed in **Supplementary Tables 3**, **4** and **Supplementary Figure 5**.

To examine protein changes in response to cold storage, samples collected at harvest were compared with control-untreated samples just after cold storage. As a result of cold, the levels of 62 proteins changed (**Supplementary Figure 3**; **Supplementary Table 3B**). These proteins are mainly located to cytosol (22.8%), cytoplasm (13.9%), chloroplast (13.9%), and mitochondrion (11.4%) (**Supplemental Figure 3**). Out of them, 26.6% pertain to metabolism, 22.8% to disease/defense, and 17.7% to energy (**Supplementary Figure 3**).

**Figure 3:**
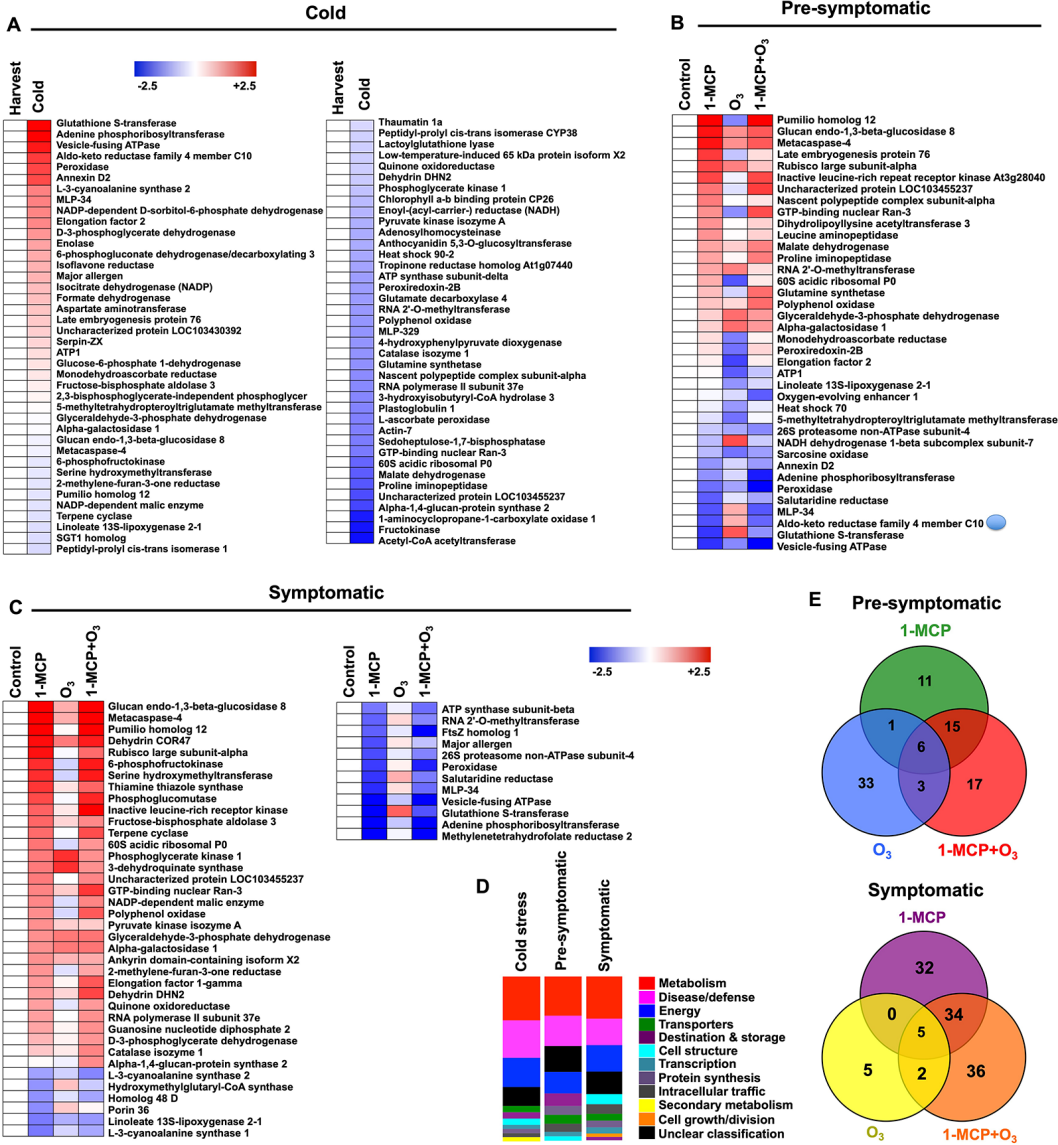
Protein hallmarks in apple peel challenged with 1-MCP and O_3_. Changes in protein accumulation patterns in apple peel exposed to 1-MCP and O_3_ treatments in response to “cold” **(A)** as well as at “presymptomatic” **(B)** and “symptomatic” period **(C)**. The heat map profiles depict the relative abundance of each protein either just after cold storage compared to harvest (for cold-affected proteins) or in 1-MCP and O_3_-treated samples compared to the abundance of untreated control (for proteins affected at “presymptomatic” and “presymptomatic” period). Fold change values are calculated as log2 ratio and were shown on a color scale, which is proportional to the abundance of each identified protein. Relative values for each protein abundance are provided in **Supplementary Table 2**. Functional categorization **(D)** of the identified proteins represented as a relative ratio proportional to the total number of the identified proteins that changed at each time (cold, presymptomatic, symptomatic stage). Venn diagrams illustrating the proteins that changed at presymptomatic and symptomatic period **(E)**. Additional details for each protein are given in **Supplementary Tables 3** and **4**.

Proteins that were differentially accumulated in apple peel in response to treatments at the presymptomatic and symptomatic scald stages are listed in **Figure 3**. At presymptomatic period, proteins can be classified into three groups (**Figure 3B**): (i) a first group represented by 33 proteins whose abundance was altered only by 1-MCP; (ii) a second group containing 43 proteins whose accumulation was modified by the O_3_; and (iii) a third group consisting of 41 proteins whose accumulation level changed following the combined application of 1-MCP and O_3_ (**Figure 3B**). Among the groups of proteins showing significant modifications in their abundance at the presymptomatic period there were six that were commonly accumulated during all treatments. The identified proteins at presymptomatic period are mainly associated with metabolism (23.7%) followed by disease/defense (18.4%) and energy (13.2%) (**Figure 3D**). They are mainly located at chloroplast (21.1%), cytosol (15.8%), and mitochondrion (10.5%) (**Supplementary Figure 3**).

At the symptomatic stage, the 1-MCP+O_3_-responsive proteins could further be grouped as follows: a group of 71 proteins whose abundance was changed exclusively by 1-MCP; (ii) a second group of 12 proteins whose abundance was changed by O_3_; and a third group of 77 proteins accumulating under the 1-MCP+O_3_ combined treatment (**Figure 3C**). Identified proteins at the symptomatic stage are involved in metabolism (26%), disease/defense (16%), and energy (16%) (**Figure 3D**). They are mainly localized to cytosol (22%) and chloroplast (16%) (**Supplemental Figure 3**). Five of them were commonly affected by all treatments (**Figure 3E**).

### Global Transcriptome Analysis Related to 1-MCP and O_3_ Function

**Figure 4:**
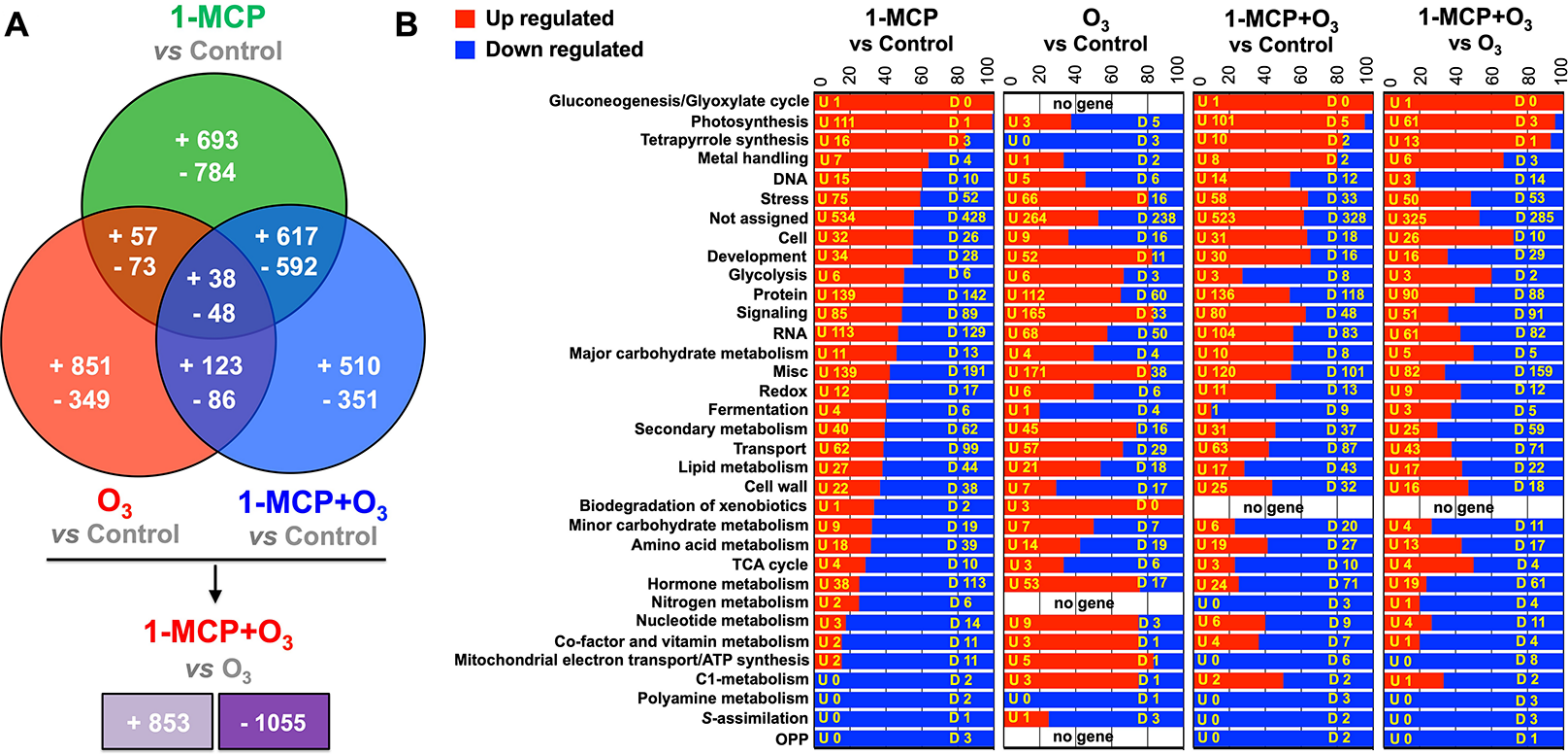
Global gene expression analysis in apple peel exposed to 1-MCP and O_3_. Venn diagram representing the transcripts that upregulated and downregulated in apple peel tissues exposed to 1-MCP and O_3_ at “presymptomatic period” (0 d at room temperature following cold) **(A)** and functional categorization of the identified transcripts that modified among the microarray comparisons **(B)**. “U” is related to the number of genes that upregulated between the 1-MCP and O_3_-treated fruits and control, while “D” is related to the number of genes that downregulated at each comparison. Additional details for each transcript are provided in **Supplementary Table 5**.

The transcriptomes of apple peel at the presymptomatic stage were analyzed to investigate early events preceding scald development. Data revealed that 6,892 transcripts were differentially expressed between treated and untreated apples (**Figure 4A**; **Supplementary Table 5**). A second analysis revealed that 1908 transcripts were regulated between O_3_ and 1-MCP+O_3_ (**Figure 4A**; **Supplementary Table 5**). Microarray data were also validated by RT-qPCR, on a subset of differentially expressed genes. Expression profiles assessed with microarrays or RT-qPCR were similar (R^2^ = 0.92 between LogRatio T/C and –DDCt; **Supplementary Figure 4**; **Supplementary Table 6**).

Based on gene annotations, transcripts were classified into 33 functional categories (**Figure 4B**). Most of the identified transcripts were closely related to photosynthesis, especially following 1-MCP treatments, in which 99.2% of these genes were found to be upregulated. Furthermore, genes that are related to stress induction, protein accumulation, signaling, RNA, transport, and hormone metabolism were also affected (**Figure 4B**). Particularly, 1-MCP led to the alteration of the expression profile of 2,902 genes, of which 1,405 were upregulated, and 1,497 were downregulated. Furthermore, O_3_ regulated 1,625 transcripts, of which 1,069 induced and 556 reduced their expression level compared to control. The third comparison between 1-MCP+O_3_ and control involved 2,365 differentially expressed genes, of which 1,288 were upregulated and 1,077 were downregulated (**Figure 4**). Furthermore, 86 transcripts (38 upregulated and 48 downregulated) were affected by all treatments (**Figure 4A**). Finally, a comparison was performed between apples in O_3_-enriched atmosphere versus 1-MCP+O_3_ treatment. In this comparison, 1908 genes were differentially expressed, of which 853 were induced in 1-MCP+O_3_ condition, while 1,055 were repressed, respectively (**Figure 4**).

### Analysis of Regulatory Networks and Modules in Apple Exposed to 1-MCP and O_3_

To characterize the mechanisms underlying scald, the relationship between regulatory molecules, transcription factors (TFs) and downstream regulated genes was investigated, using expression data for genes and TFs incorporating protein and metabolite data. The output of a regulatory program is rarely due to the action of a single gene, being rather the result of the cooperation of multiple genes and their common regulation under the same TFs that result in the desired output. Notably, a group of coexpressed genes comprises a single module as well as the whole entire set of regulatory interactions between the TFs and the modules. Expression profiling was used to infer the regulatory networks that comprise regulated genes organized in modules as well as the TFs regulating these modules. The output of the LeMoNe algorithm is a set of modules of coexpressed transcripts, providing a list of high-scoring TFs regulating the modules that were prioritized according to their corresponding scald syndrome. The algorithm assigns sets of TF regulators to each of the modules using a probabilistic scoring, taking into account the profile of the candidate regulator. Genes were first clustered into 235 modules (**Supplementary Table 7**) of which 22 were regulated by one or more TFs (**Supplementary Table 7**). This resulted in 46 transcription factor-module regulatory interactions that exceeded the above-random weight threshold (**Supplementary Table 7**). These TFs mainly include the following families: ARF (auxin response factors), bHLH (basic helix-loop-helix proteins playing a major role in jasmonates signaling), bZIP (basic leucine zipper proteins involved in photomorphogenesis, leaf and seed formation, energy homeostasis, and abiotic and biotic stress responses), CO-like (product of the CONSTANS gene acting between the circadian clock and genes controlling meristem identity), ERF (ethylene response factors coordinating stress signaling), FAR1, GRAS (named after the three members: GIBBERELLIC-ACID INSENSITIVE, REPRESSOR of GAI and SCARECROW, these TFs are involved in plant growth and development, HD-ZIP (homeodomain-leucine zipper involved in plant development and morphogenesis as well as responses to stresses), LBD (lateral organ boundary domain, acting as negative regulators of anthocyanin biosynthesis in Arabidopsis), MADS (MCM1, AGAMOUS, DEFICIENS, and SRF (serum response factor) TFs, involved in many aspects of plant development), MYB (myeloblastosis family of TFs acting as key factors in regulatory networks controlling plant development, metabolism and responses to stresses), NAC (named from the three genes: NAM, ATAF1 and −2, and CUC2, these TFs are involved in the regulation of the transcriptional reprogramming associated with stress responses), NF-YA (NUCLEAR FACTOR-Y A, involved in plant growth and development), S1Fa (which specifically recognizes a negative element (S1F) within the *RPS1* promoter), TALE (transcription activator-like effector), TCP (plant-specific TEOSINTE BRANCHED 1, CYCLOIDEA, PCF1 TF family), Trihelix (TFs carrying three α-helical sequences separated by loops or turns), WOX (plant-specific *WUSCHEL-related homeobox* TF gene family), YABBY (plant-specific TFs with zinc finger and high mobility group-related domains, involved in the specification of abaxial polarity in lateral organs) and ZF-HD (zinc finger homeobox family protein) (**Supplementary Table 7**).

### Key Changes in TFs-Gene-Protein-Metabolite Interactome Following 1-MCP and O_3_ Application

Following the above network analysis, we examined the TFs-gene-protein-metabolite shift that was strongly affected by 1-MCP and O_3_ treatments (**Figure 6**). One of the main highlighted relationships shared between the two chemical treatments was the increase in *PPO* gene (*MDP0000699845*) was coregulated with HD-ZIP and encode PPO protein (**Figure 6**; **Supplementary Table 7**). Additionally, TFs and related transcripts for five putative proteins, namely glucan glucosidase 8, 6-phosphofructokinase, FtsZ homolog 1, ATP synthase subunit-beta and lipid-transfer protein were positively or negatively affected by treatments (**Figure 6**). A notable feature of our analysis was that a large number of TFs *(HD-ZIP, FAR1, ZF-HD, NF-YA, MYB, S1Fa-like, YABBY, and BHLH) along with* downstream target glutathione *S*-transferase (GSTs) genes (*MDP0000178304*, *MDP0000256360*, *MDP0000252292*, *MDP0000805474*, *MDP0000766223*, *MDP0000261432*, *MDP0000722969*, and *MDP0000566567*) and corresponding GSTs proteins showed the most striking treatment-specific regulation (**Figure 6**; **Supplementary Table 7**), indicating that GSTs play a key role in scald.

## Discussion

Despite the economical importance of superficial scald in apple, the etiology and biochemistry that lead to the development of this disorder are not completely understood (Lurie and Watkins, 2012; Storch et al., 2017). In this study, we found that fruits exposed to 1-MCP exhibited inhibition of ethylene-dependent ripening processes (**Figures 1C–G**; **Supplementary Figure 1**) and displayed considerably reduced scald symptoms (**Figures 1A, B**). We previously found that kiwifruit ripening and ethylene production were depressed by O_3_ (Minas et al., 2014; Minas et al., 2018). In marked contrast with the situation observed with kiwifruit, the ripening behavior (**Figures 1C–G**) as well as the activities of ACS and ACO, and the ACC and MACC levels along with ethylene production (**Figures 1C–G**) remained unaffected in O_3_-treated apples (**Supplementary Figure 1**). This indicates that the ripening effects of O_3_ are different depending on the fruit species and/or tissue analyzed. However, the data from the current study point to *a novel observation*, namely that O_3_ noticeably induced visible scald injury in apple (**Figures 1A, B**). Another important result revealed by this study was that fruits subjected to 1-MCP in the presence of O_3_ exhibited scald-healthy phenotypes (**Figures 1A, B**), confirming that 1-MCP is a very efficient tool to prevent apple scald (Lurie and Watkins, 2012). Such distinct 1-MCP- and O_3_-driven scald features (**Figures 1A, B**) provide a relevant model for exploring the mechanisms associated with apple cold responses as well as with scald prevention/sensibility.

Long term cold exposure induced major metabolic and biochemical *changes* in fruits (Molassiotis et al., 2013). Accordingly, we observed a strong reduction in several amino acids, such as aspartic acid, glutamic acid, serine, threonine, and valine in apple peel following cold storage (**Figure 2**). Proteins coding numerous antioxidant-related enzymes (e.g., peroxidase and monodehydroascorbate reductase, and NADPH-generating systems (e.g., 6-phosphogluconate dehydrogenase) along with energy-associated enzymes (e.g., adenine phosphoribosyltransferase, ATP1 and vesicle-fusing ATPase) were induced by cold (**Figure 3A**; **Supplementary Table 3B**). Also, various ripening enzymes (e.g., ACO, malate dehydrogenase, and fructokinase) were repressed by cold (**Figure 3A**; **Supplementary Table 3B**).

Metabolome data indicated that unlike O_3_ application, the 1-MCP treatment provoked the accumulation of several amino acids both before and during scald. Our results, when taken together with previous ones (Osuna et al., 2007; Less and Galili, 2008), suggest important roles for amino acid biosynthesis in scald responses. In this regard, an intriguing observation was the greater accumulation of threonine and valine in 1-MCP-treated compared to O_3_-treated fruits (**Figure 2**), particularly indicating that different scald phenotypes may reflect specific reprogramming in branched-chain amino acids (BCAAs). It has been shown that BCAAs accumulation may serve as a substrate for the synthesis of stress-induced proteins and that BCAAs may act as signaling molecules to regulate gene expression in response to cold (Goddard et al., 1993). In agreement with recent data obtained in peach fruit showing that BCAAs accumulation together with the upregulation of genes encoding BCAAs-rich proteins confer tolerance against cold injury (Tanou et al., 2017). Furthermore, steady-state levels of serine exhibited distinct patterns in 1-MCP and O_3_ treated samples (**Figure 2**). We have shown that protein abundance of D-3-phosphoglycerate dehydrogenase (PHGDH) and serine hydroxymethyltransferase (SHMT) was induced by both 1-MCP treatments but depressed by O_3_ alone application (**Figure 3C**; **Supplementary Table 3**). PHGDH catalyzes the transition of 3-phosphoglycerate into 3-phosphohydroxypyruvate, which is the committed step in the phosphorylated pathway of L-serine biosynthesis while SHMT catalyzes the reversible interconversion of serine and glycine with tetrahydrofolate serving as the one-carbon carrier. Given the role of serine in photorespiration in mitigating production of ROS at the chloroplast (Leegood et al., 1995), the observed stimulation of serine metabolism along with the upregulation of various photosynthetic genes by 1-MCP (**Figure 4B**) might result in an activation of the antioxidant defenses leading to a reduced level of chloroplast oxidation and reduced scald susceptibility. Consistent with this possibility, photorespiratory *Arabidopsis* mutants specifically defective in SHMT showed spontaneous formation of chlorotic and necrotic lesions (Moreno et al., 2005).

A set of apple peel proteins displayed high accumulation levels in healthy fruits (1-MCP treated), both at presymptomatic and especially at symptomatic periods, whereas O_3_-treated fruits had decreased scald symptoms (**Figures 3A, B**). This implies, quite remarkably, that a specific 1-MCP and O_3_-specific proteome remodeling occurs that could be correlated with scald phenotypes. Posttranscriptional/translational control of gene expression is a powerful strategy for fruit cells to adapt to postcold ripening period as this process is controlled by various RNA-binding proteins (Huh and Paek, 2014). At pro-symptomatic and symptomatic periods, we observed in 1-MCP exposed fruits an increased abundance of Pumilio 12 (*PUM12*) (**Supplementary Tables 3C, D**), a protein that binds to sequences in the 3′ UTRs of target mRNAs through their PUMILIO homology domains, suggesting a correlation between *PUM12* accumulation and scald prevention. This protein would participate in scald response through binding to specific regulatory *cis*-elements of their mRNA targets, to regulate RNA translation (Lee et al., 2016) and thus involved in protein synthesis. There are examples in plants indicating that nucleo-cytoplasmic trafficking of proteins can also affect posttranscriptional regulation, such as mRNA processing and RNA trafficking (Wang et al., 2010). Interestingly, we found that the abundance of the GTP-binding nuclear protein Ran-3, which is required for nucleo-cytoplasmic protein/RNA partitioning, along with the abundance of elongation factor 2 (eEF2; a GTP-binding protein), was both higher in 1-MCP-treated than in O_3_-scalded fruits (**Supplementary Table 3**). It is thus likely that scald prevention is associated with nucleo-cytoplasmic transport of proteins and RNA, possibly because this mechanism allows for quick responses from cold to higher temperature transition without *de novo* protein and RNA syntheses. Such a mechanism seems to be impaired in fruits exposed to the single O_3_ treatment, since we found, for example, that the accumulation of the Ran-3 protein was suppressed by O_3_ (**Supplementary Table 3**).

Among the multiple molecular mechanisms underlying cold responses, alterations of the protein translation machinery affecting the rate and selectivity of stress-related protein biosynthesis may play a central role (Wang et al., 2017). The higher accumulation of the 60S acidic ribosomal protein P1, involved in the elongation step of protein synthesis, observed at both pro-symptomatic and symptomatic time following 1-MCP application (**Supplementary Tables 3C, D**) should contribute to scald prevention. Noticeably in the context of this work, Nakaminami et al. (2014) showed that the 60S acidic ribosomal protein P1 is involved in cold acclimation in Arabidopsis. Exposure to O_3_ alone suppressed the accumulation of several stress-related proteins, such as the late embryogenesis protein 76, peroxiredoxin-2B and monodehydroascorbate reductase (**Supplementary Table 3**), which may well explain the aberrant scald defect in O_3_-treated apple (**Figures 1A, B**).

A large number of apple genes exhibited 1-MCP and O_3_-associated patterns of expression (**Figure 4**) that could possibly affect scald responses (**Figures 1A, B**). We focused on the genes that displayed opposite distinct patterns of regulation between 1-MCP and O_3_. In this set of genes we found a strong induction of various photosynthetic genes only following 1-MCP treatments (**Figure 4**). This fact fits well with the 1-MCP accociated retention of green color in the peel of ‘Granny Smith’ apples (**Figure 1A**), suggesting that the photosynthetic machinery could be accociated with scald. This 1-MCP-*exclusive photosyntetic group* includes genes annotated as photosystem I subunit D-2 (*PSAD-2; MDP0000700880*), photosystem I reaction center subunit PSI-N (*PSAN; MDP0000655277*), photosystem II subunit P-1 (*PSBP-1; MDP0000361338*), PsbQ-like 2 (*MDP0000481445*), photosystem II subunit Q-2 (*PSBQ/PSBQ-2; MDP0000715912*) rubisco activase (RCA; *MDP0000223905*), ribulose bisphosphate carboxylase (RBC; *MDP0000185022*), and ribulose bisphosphate carboxylase (small chain) protein (*MDP0000185022*) (**Supplementary Table 5**). The upregulation of photosynthetic genes (**Figure 4**) could be linked to the inhibition of ethylene synthesis and signaling by 1-MCP, which in turn might increase the efficiency of ROS-scavenging system (Foyer and Shigeoka, 2010), leading to lower oxidative stress and scald prevention. We noted that the expression of several stress-related transcripts, including those encoding leucine-rich repeat protein (*MDP0000331536*), HVA22 homologue D (*MDP0000574524*; homologous to a eukaryote specific ABA- and stress-inducible gene first isolated from barley) and HSP20-like chaperones (*MDP0000574524*) were solely induced by 1-MCP (**Supplementary Table 5**). Given that these genes are involved in cold acclimation (Shen et al., 2001; Lopes-Caitar et al., 2013; Liao et al., 2017), our data suggests that the induction of stress-related gene expression by 1-MCP could contribute to apple fruit tolerance against cold stress. In addition, 1-MCP repressed several ethylene signaling genes, such as ethylene response sensor 1 (ERS1; *MDP0000242413*) (**Supplementary Table 5**). We also observed a strong downregulation of various genes only by 1-MCP that could be directly linked to scald. An interesting example is the downregulation of the gene encoding farnesyl diphosphate synthase 2 (FPS2; *MDP0000198736*) by 1-MCP, whose expression was found to be tightly linked to scald syndrome (Tsantili et al., 2007; Karagiannis et al., 2018b), supporting the present scald characterization.

The expression of several transcripts was exclusively affected by O_3_ and therefore might affect scald development (**Figure 4**). In particular, O_3_ appeared to upregulate receptor-like proteins 15 (RLP, an important class of cell-surface receptors; *MDP0000302222*) and naringenin-chalcone synthase (CHS, the first committed enzyme in flavonoid biosynthesis; *MDP0000432621*) (**Supplementary Table 5**). Studies on several RLPs genes have revealed gene expression changes, as well as the emergence of phenotypic alterations, following application of specific elicitors (Wang et al., 2010). This result highlights that O_3_-enriched cold storage atmosphere stimulated RLP15 expression, presumably because of O_3_ action as a signal molecule for the activation of cell-surface receptors to perceive O_3_-specific signals. In the flavonoid pathway CHS catalyzes the stepwise condensation of three acetate residues from malonyl-CoA with the phenylpropanoid biosynthetic intermediate *p*-coumaroyl CoA to form naringenin chalcone. Naringenin-chalcone synthase was previously shown to be O_3_-sensitive on the basis of the appearance of macroscopic injury in plant cells (Paolacci et al., 2002). This supports the possibility that susceptibility to scald might rest on a sequence of molecular events similar to that leading to programmed cell death (PCD), which was indirectly observed as one of the scald symptoms (Busatto et al., 2014; Karagiannis et al., 2018b).

**Figure 5:**
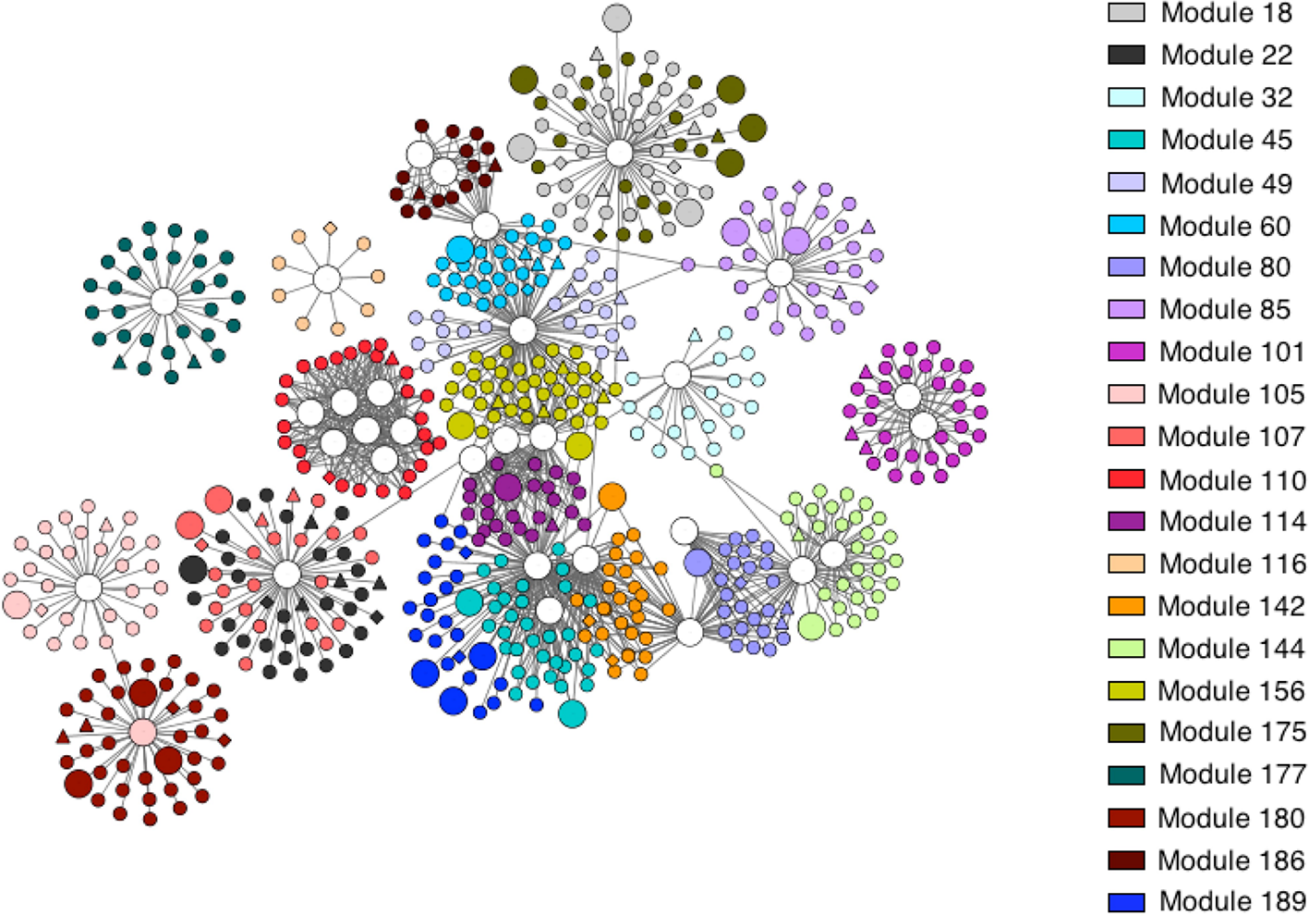
Transcriptional regulatory network in apple peel at pro-symptomatic period. Nodes represent genes, edges regulatory interactions between a transcription factor and a gene. Triangle-shaped nodes represent nodes with significant protein expression; diamond-shaped nodes represent genes with significant metabolite expression; larger circles represent transcription factors. Modules are color-coded as indicated in the inset.

**Figure 6:**
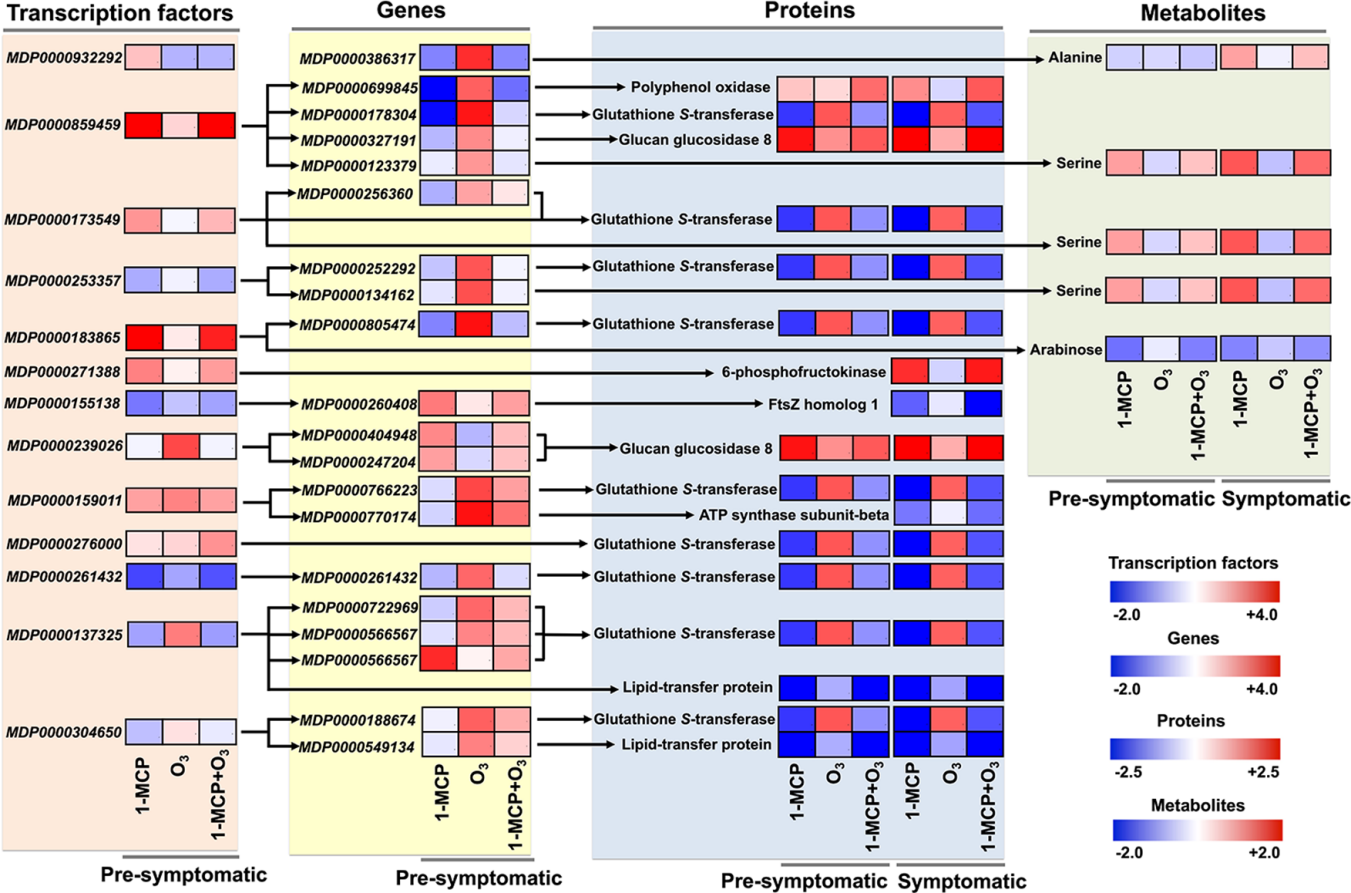
Key TFs-gene-protein-metabolite interactome shift that affected by 1-MCP and O_3_. Schematic representation showing a TFs-gene-protein-metabolite interactome shift, based on network analysis, that was remarkably altered in response to chemical treatments. The heat maps represent the relative level of each component at presymptomatic and symptomatic stage. The color scales that are proportional to the ratio of each identified component shows the fold change between treated and control fruits. Relative expression data are shown in **Supplementary Tables 1**, **2**, and **5**. *MDP0000386317: ALD1 (AGD2 - LIKE DEFENSE RESPONSE PROTEIN1); transaminase, MDP0000699845: polyphenol oxidase, MDP0000178304: ATGSTU8 (Arabidopsis thaliana glutathione S-transferase (class tau) 8); glutathione transferase, MDP0000327191: BG3 (BETA-1,3-GLUCANASE 3); hydrolase, hydrolyzing O-glycosyl compounds, MDP0000123379: protein kinase family, MDP0000256360: ATGSTF8 (glutathione S-transferase 8); glutathione S-transferase, MDP0000252292: ATGSTF12 (glutathione S-transferase 26); glutathione transferase, MDP0000134162: RLK1 (RECEPTOR-LIKE PROTEIN KINASE 1); carbohydrate binding/kinase, MDP0000805474: ATGSTU8 (Arabidopsis thaliana glutathione S-transferase (class tau) 8); glutathione transferase, MDP0000260408: transducin family protein/WD-40, MDP0000404948: glycosyl hydrolase family 17 protein, MDP0000247204: glycosyl hydrolase family 17 protein, MDP0000766223: ATGSTU8 (Arabidopsis thaliana glutathione S-transferase (class tau) 8); glutathione transferase, MDP0000770174: PAD4 (PHYTOALEXIN DEFICIENT 4); triacylglycerol lipase, MDP0000261432: plant-specific YABBY family protein, MDP0000722969: ATGSTU25 (Arabidopsis thaliana glutathione S-transferase (class tau) 25); glutathione transferase, MDP0000566567: ATGSTU21 (Arabidopsis thaliana glutathione S-transferase (class tau) 21); glutathione transferase, MDP0000188674: In2-1 protein, putative, MDP0000549134: protease inhibitor/seed storage/lipid transfer protein (LTP)*.

To characterize the mechanism of scald development, the transcript, protein and metabolite data sets were integrated using bioinformatics tools to identify TFs regulating gene modules with similar coexpression patterns (**Figure 5**). Based on network analysis, we constructed an integrated analysis of TFs-gene-protein-metabolite data sets and we focused on certain important interactome shift that dispayed redundant changes in response to appied treatments (**Figure 6**). Using this approach, we observed that PPO was strongly affected by treatments, which is consistent with its effect on scald-derived browning hallmarks in apple skin (Boss et al., 1995; Mayer, 2006; Busatto et al., 2014). Our analysis identified that genes coregulated by TFs encoding nine GTS proteins disclosed completely distinct regulation in response to 1-MCP and O_3_ (**Figure 6**). Recent research also uncovered an important role of GSTs in scald development (Du et al., 2017; Karagiannis et al., 2018b; Wang et al., 2018) thereby pointing out these enzymes as promising candidates for further functional characterizations. What then might be the role of GSTs in scald physiology? It is possible that some apple GSTs may participate in the detoxification of reactive aldehydes and related compounds that are generated by the loss of membrane integrity during scald development (Busatto et al., 2018). We found that several other aldehyde scavengers, such as aldo-keto reductase family 4 member C10 and glyceraldehyde-3-phosphate dehydrogenase, were stimulated by O_3_ at pro-symptomatic period (**Figure 3**). The idea that proper lipid modification is needed to activate the detoxification of lipid peroxidation-produced toxic compounds is also supported by the observed similar expression pattern of GSTs with the lipid-transfer proteins (LTPs) (**Figure 6**), which are required for assembly of the water-proof lipid barriers—such as cutin and cuticular wax and suberin—largely present on apple peel surface (Beisson et al., 2012). Particularly, we observed that LTP *(MDP0000722969* and *MDP0000188674)* and GSTs (encoded by *MDP0000566567 and MDP0000549134)* were commonly regulated by basic helix-loop-helix (bHLH) TF, suggesting that this TF could be critical for scald development. Feng et al. (2012) also showed that the apple bHLH gene *MdCIbHLH1* (*Cold-Induced bHLH1*) was induced in response to cold stress. It has been demonstrated that the MdCIbHLH1 protein bound to the promoters of *MdCBF2* and favorably contributed to cold tolerance in transgenic apple plants by upregulating the expression of *MdCBF2* through the ICE-CBF/DREB1 pathway (Feng et al., 2012). It is widely recognized that the ICE (Inducer of CBF Expression)-C‐repeat binding factor (CBF) transcriptional regulatory cascade globally regulates cold-response in *Arabidopsis* (Knight and Knight, 2012). There is strong evidence that ICE1, a bHLH transcription factor, directly binds to *cis*-elements (CANNTG) in the *CBF3/DREB1a* promoter (Chinnusamy et al., 2003), thereby linking bHLH with the ICE-CBF/DREB1 cold pathway. In parallel, ethylene signaling was shown to negatively regulate plant cold stress responses by repressing the ICE-CBF/DREB1 system in *Arabidopsis* (Shi et al., 2012). This link between ethylene and postcold apple metabolism is further supported by the identified function of two ERF TFs (encoded by *MDP0000932292* and *MDP0000127134*) toward 1-MCP and O_3_ treatments (**Supplementary Table 7**). An alternative possibility to account for the observed transcriptionally upregulation of GST in all scald-affected circumstances (control and O_3_) could be that GST is induced as a consequence of cold stress and scald expression. It has been reported that the levels of glutathione (GSH), together with the GSH metabolite cysteinil-glycine, were increased in stressed ‘Granny Smith’ fruits undergoing scald while decreased in 1-MCP treated fruits (Zermiani et al., 2015), suggesting that GSH cycling was regulated by scald probably to support ROS control. This hypothesis was reinforced by the profile of a plethora of ROS metabolising genes and enzymes in the scald-affected apple peel (**Supplementary Tables 4** and **5**). *Further research* is needed to reveal the exact functions of *GST in* scald.

## Conclusion

This work provides novel information as to how 1-MCP and especially O_3_ may regulate scald expression, while also offering a framework for the gene, proteins, metabolite, and transcriptional networks that are functioning during the early and late stages of scald development. Data of the experimental 1-MCP and O_3_ system described in this article supported the importance of accumulation of several amino acids, including branched-chain amino acids, as well as the nucleo-cytoplasmic transport of proteins and RNA protein translation in scald. Interestingly, the 1-MCP induced scald tolerance was correlated with the active expression of several genes in apple peel that are involved in *photosynthesis, stress responses*, flavonoid biosynthesis and ethylene signaling. We also constructed a trans-omic network of TFs using a combination of data generated in this study. This approach suggested a crucial role of PPO and especially GST protein in scald progress. Altogether, this integrated study could help to better understand regulatory mechanisms underlying scald in apple fruit.

## Data Availability Statement

Microarray data have been submitted to the Gene Expression Omnibus under the accession number GSE133456. The proteomic datasets generated for this study can be found in the ProteomeXchange accession PXD016849.

## Author Contributions

EK and AM conceived the project and its components. EK, GT, and MMi collected samples and performed physiological analysis. EK, MO, and FL conducted gene expression and data analysis. EK, GT, MS, and DJ acquired and analyzed proteomic data. FS and AF acquired and analyzed metabolic data. MMa performed bioinformatics analysis. EK and AM wrote the manuscript. All authors read, revised, and approved the final manuscript.

## Funding

The research work was partially supported by the Hellenic Foundation for Research and Innovation (H.F.R.I.) under the “First Call for H.F.R.I. Research Projects to support Faculty members and Researchers and the procurement of high-cost research equipment grant” (Project Number: 633; GERASKO).

## Conflict of Interest

The authors declare that the research was conducted in the absence of any commercial or financial relationships that could be construed as a potential conflict of interest.

The handling Editor declared a past collaboration with one of the authors AF.
