# Supplementary material for: Systems-Based Approaches to Unravel Networks and Individual Elements Involved in Apple Superficial Scald
Source: Front Plant Sci. 2020 Feb 13;11:8. doi: 10.3389/fpls.2020.00008 (PMC7031346; doi:10.3389/fpls.2020.00008)
Supplement: Supplementary file 8 [file Presentation_1.pptx]

## Slide 1
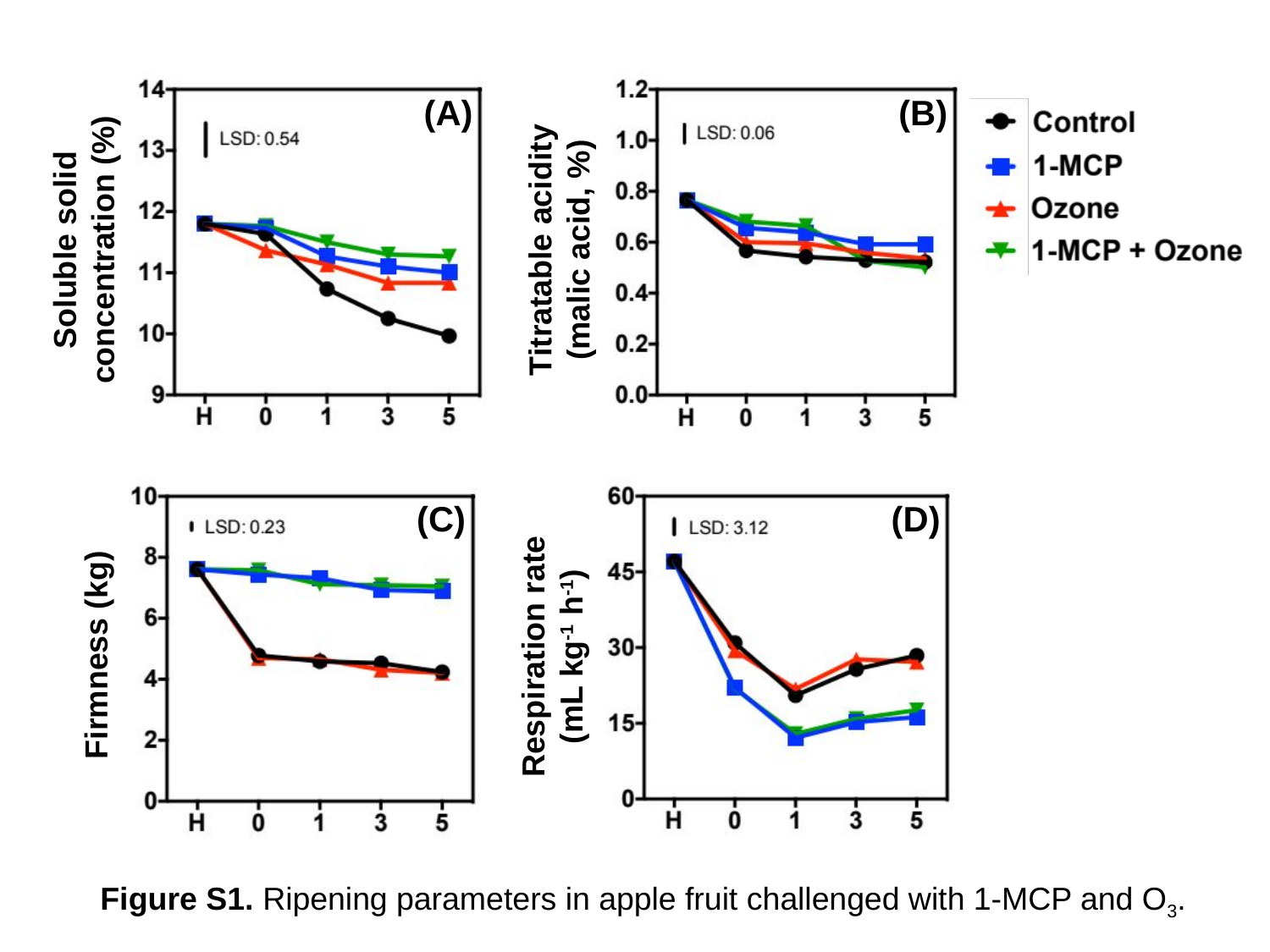

(A)
(B)
Titratable acidity (malic acid, %)
Soluble solid concentration (%)
(C)
(D)
Respiration rate
(mL kg-1 h-1)
Firmness (kg)
Figure S1. Ripening parameters in apple fruit challenged with 1-MCP and O3.
