# Supplementary material for: Systems-Based Approaches to Unravel Networks and Individual Elements Involved in Apple Superficial Scald
Source: Front Plant Sci. 2020 Feb 13;11:8. doi: 10.3389/fpls.2020.00008 (PMC7031346; doi:10.3389/fpls.2020.00008)
Supplement: Supplementary file 9 [file Presentation_2.pptx]

## Slide 1
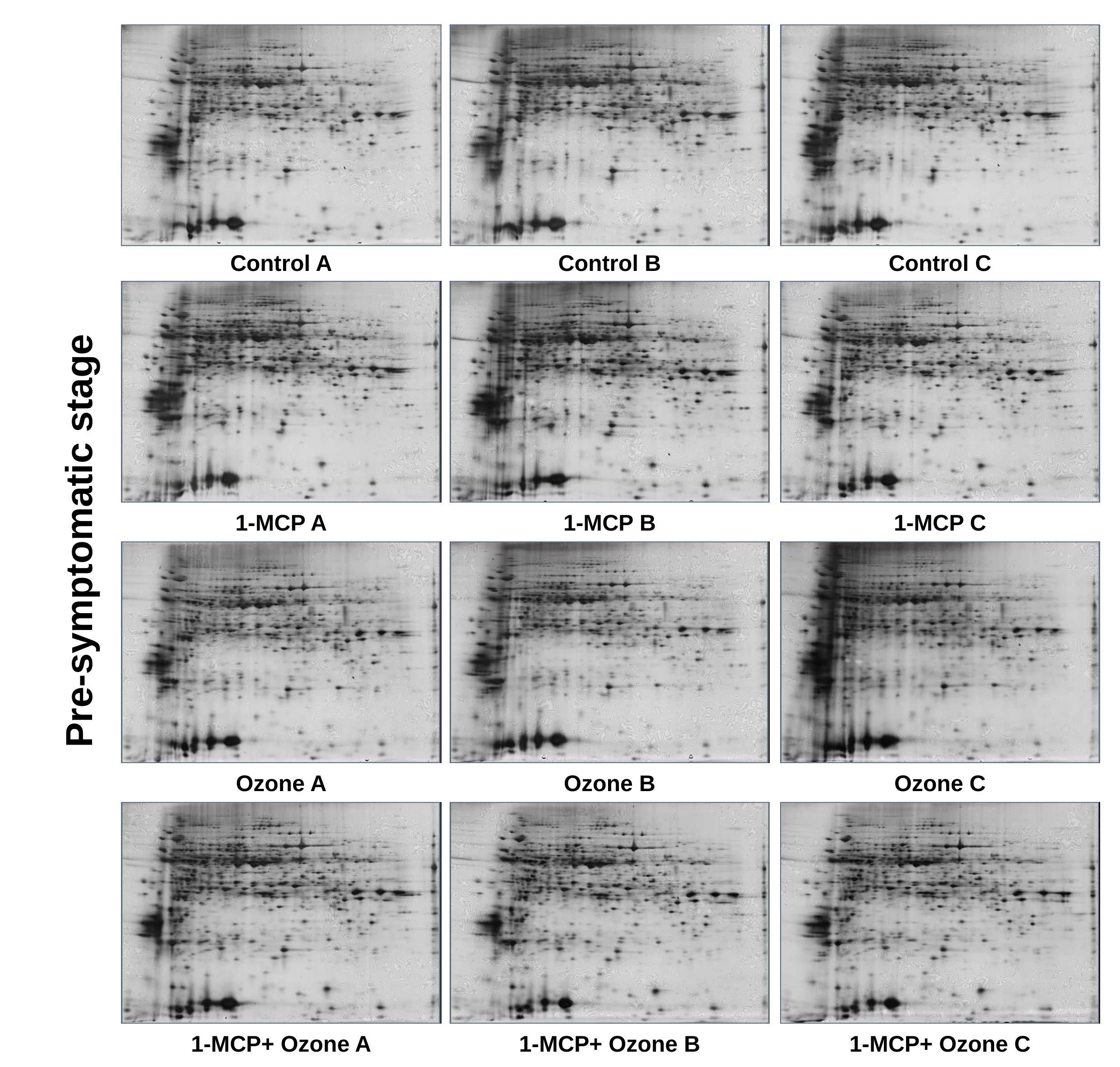

Control A
Control B
Control C
1-MCP A
1-MCP B
1-MCP C
Pre-symptomatic stage
Ozone A
Ozone B
Ozone C
1-MCP+ Ozone A
1-MCP+ Ozone B
1-MCP+ Ozone C

## Slide 2
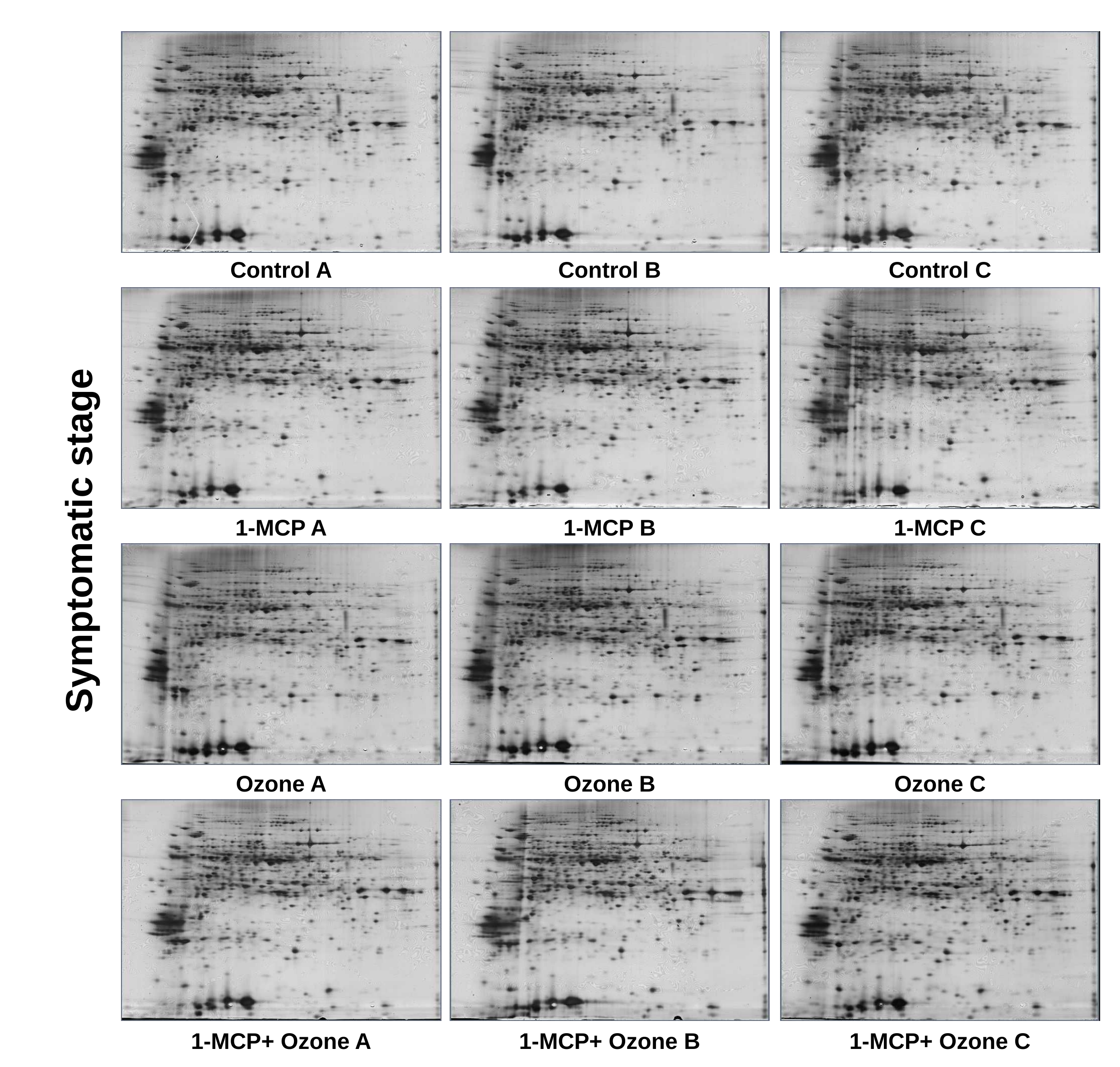

Control A
Control B
Control C
1-MCP A
1-MCP B
1-MCP C
Symptomatic stage
Ozone A
Ozone B
Ozone C
1-MCP+ Ozone A
1-MCP+ Ozone B
1-MCP+ Ozone C

## Slide 3
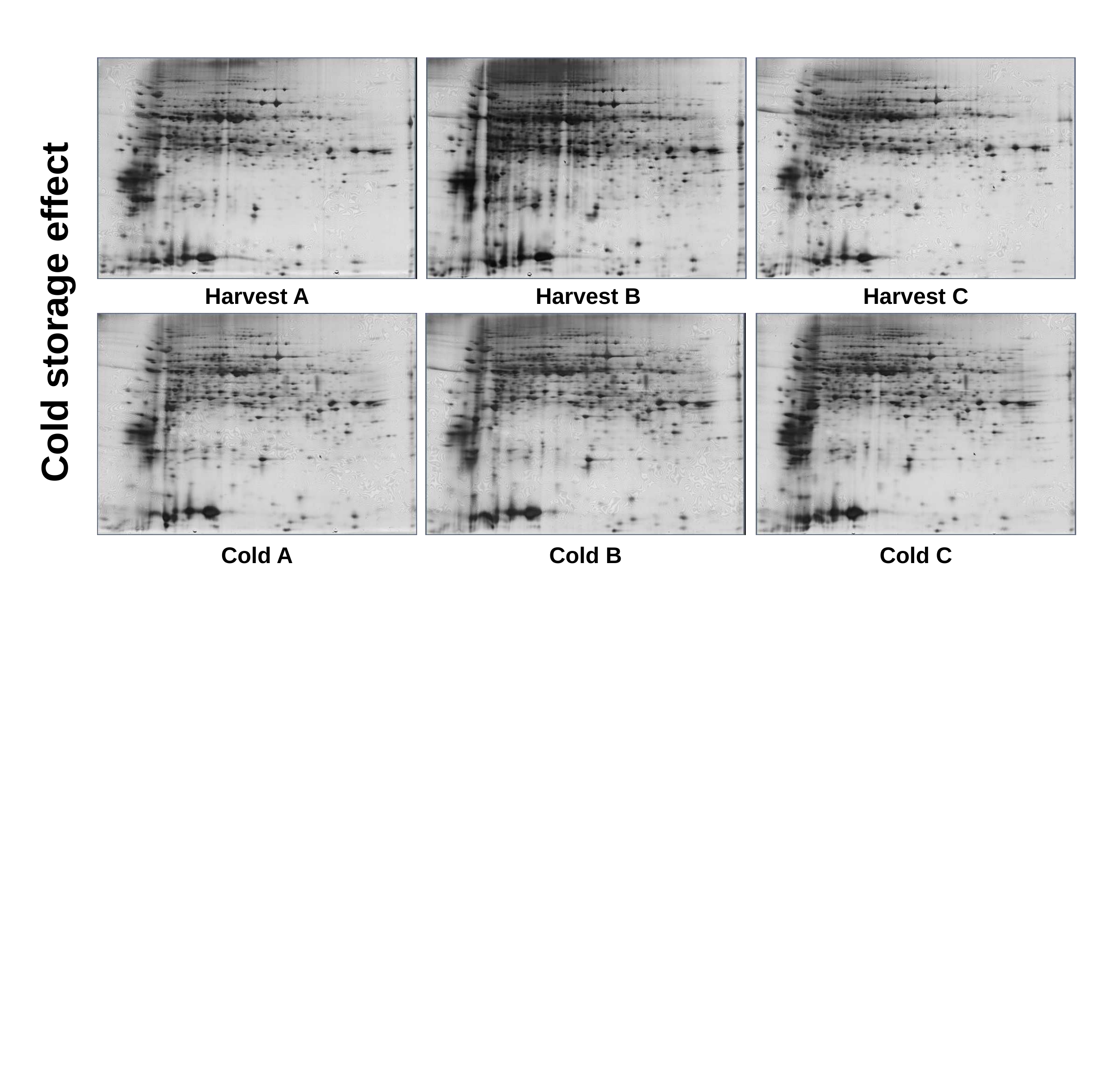

Harvest C
Harvest A
Harvest B
Cold storage effect
Cold A
Cold B
Cold C
