# Supplementary material for: Systems-Based Approaches to Unravel Networks and Individual Elements Involved in Apple Superficial Scald
Source: Front Plant Sci. 2020 Feb 13;11:8. doi: 10.3389/fpls.2020.00008 (PMC7031346; doi:10.3389/fpls.2020.00008)
Supplement: Supplementary file 10 [file Presentation_3.pptx]

## Slide 1
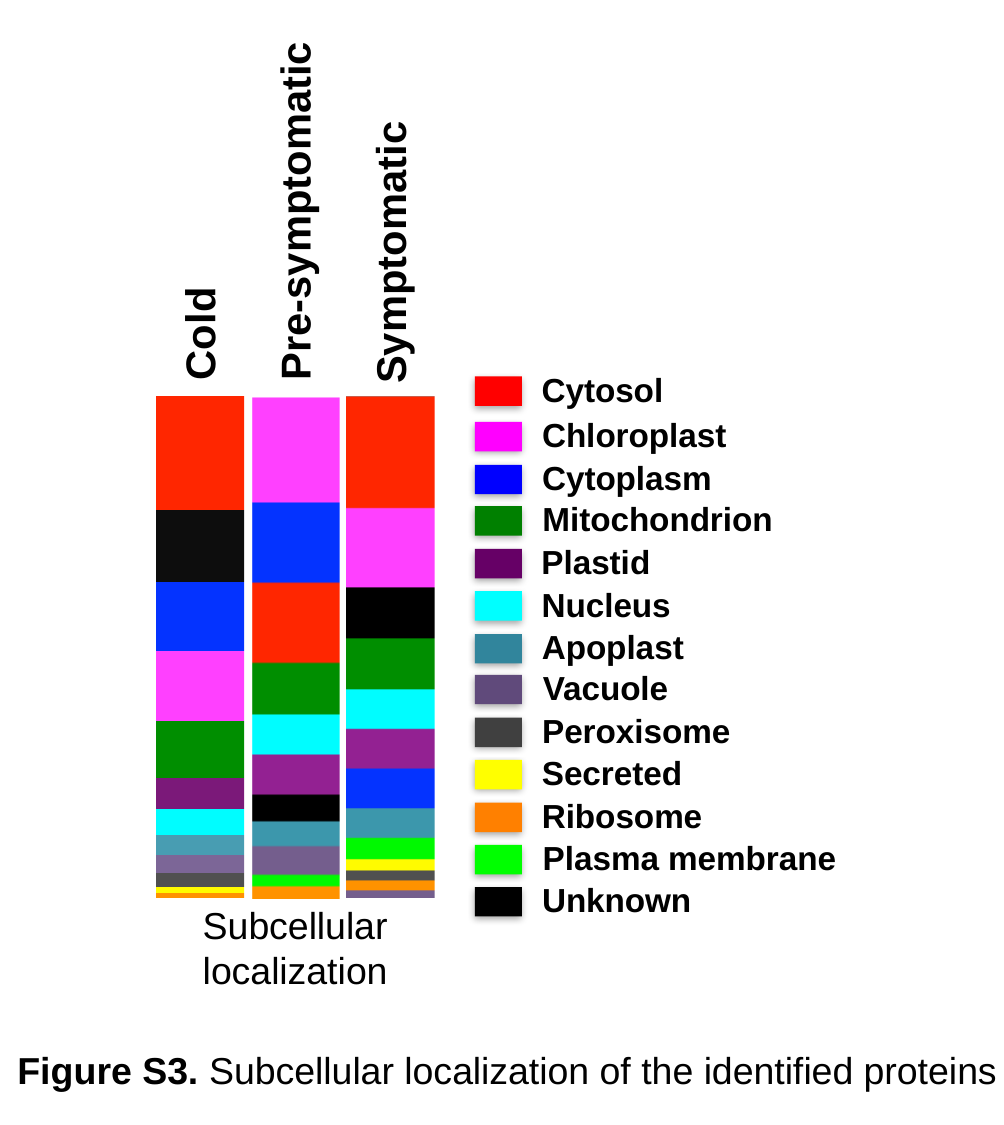

Pre-symptomatic
Symptomatic
Cold
Cytosol
Chloroplast
Cytoplasm
Mitochondrion
Plastid
Nucleus
Apoplast
Vacuole
Peroxisome
Secreted
Ribosome
Plasma membrane
Unknown
Subcellular
localization
Figure S3. Subcellular localization of the identified proteins
