# Supplementary figures and images for: Systems-Based Approaches to Unravel Networks and Individual Elements Involved in Apple Superficial Scald
Source: Front Plant Sci. 2020 Feb 13;11:8. doi: 10.3389/fpls.2020.00008 (PMC7031346; doi:10.3389/fpls.2020.00008)

## Slide 1
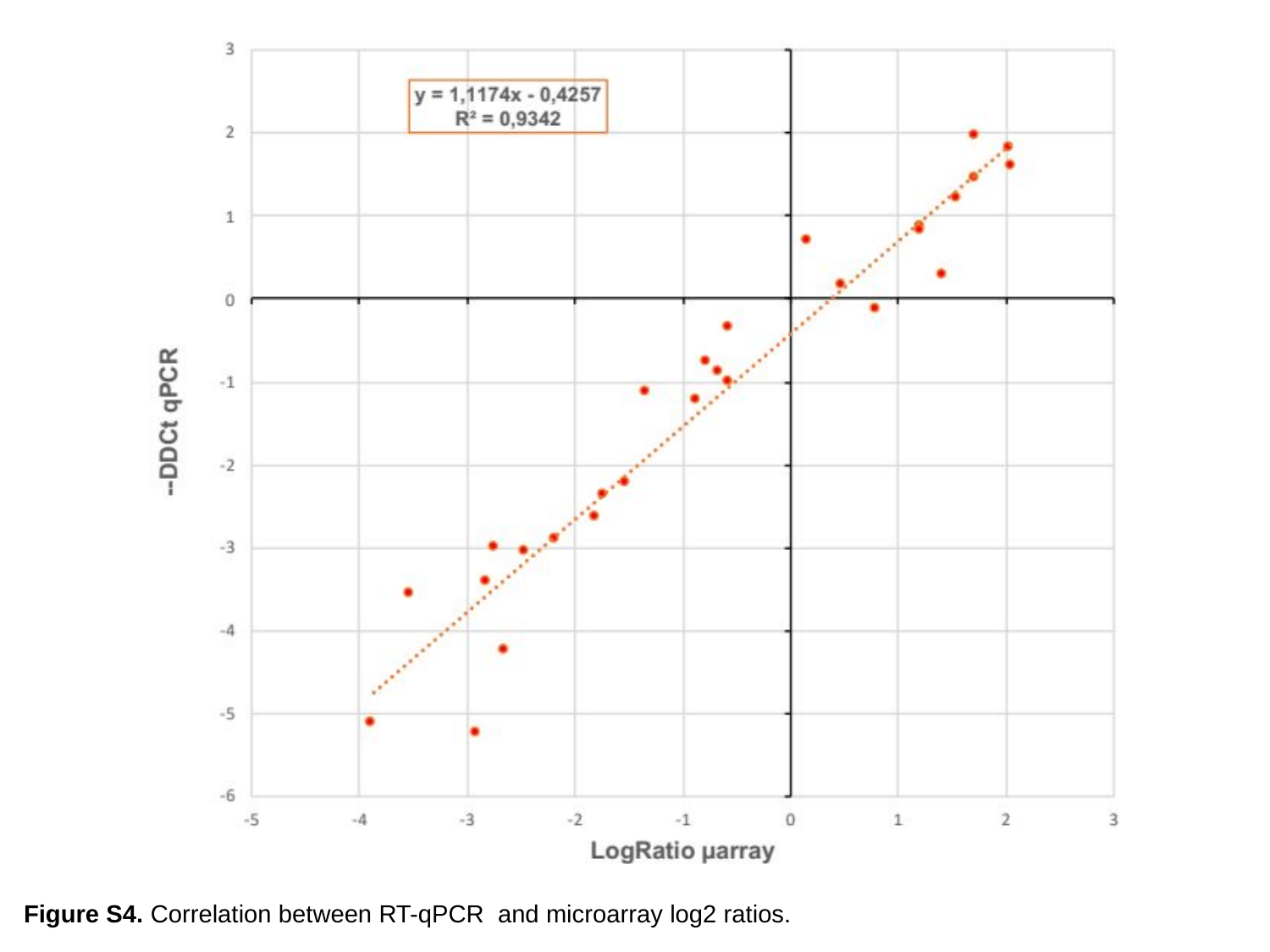

Figure S4. Correlation between RT-qPCR and microarray log2 ratios.

Supplement: Supplementary file 11 [file Presentation_4.pptx]
