# Supplementary material for: Systems-Based Approaches to Unravel Networks and Individual Elements Involved in Apple Superficial Scald
Source: Front Plant Sci. 2020 Feb 13;11:8. doi: 10.3389/fpls.2020.00008 (PMC7031346; doi:10.3389/fpls.2020.00008)
Supplement: Supplementary file 12 [file Presentation_5.pptx]

## Slide 1
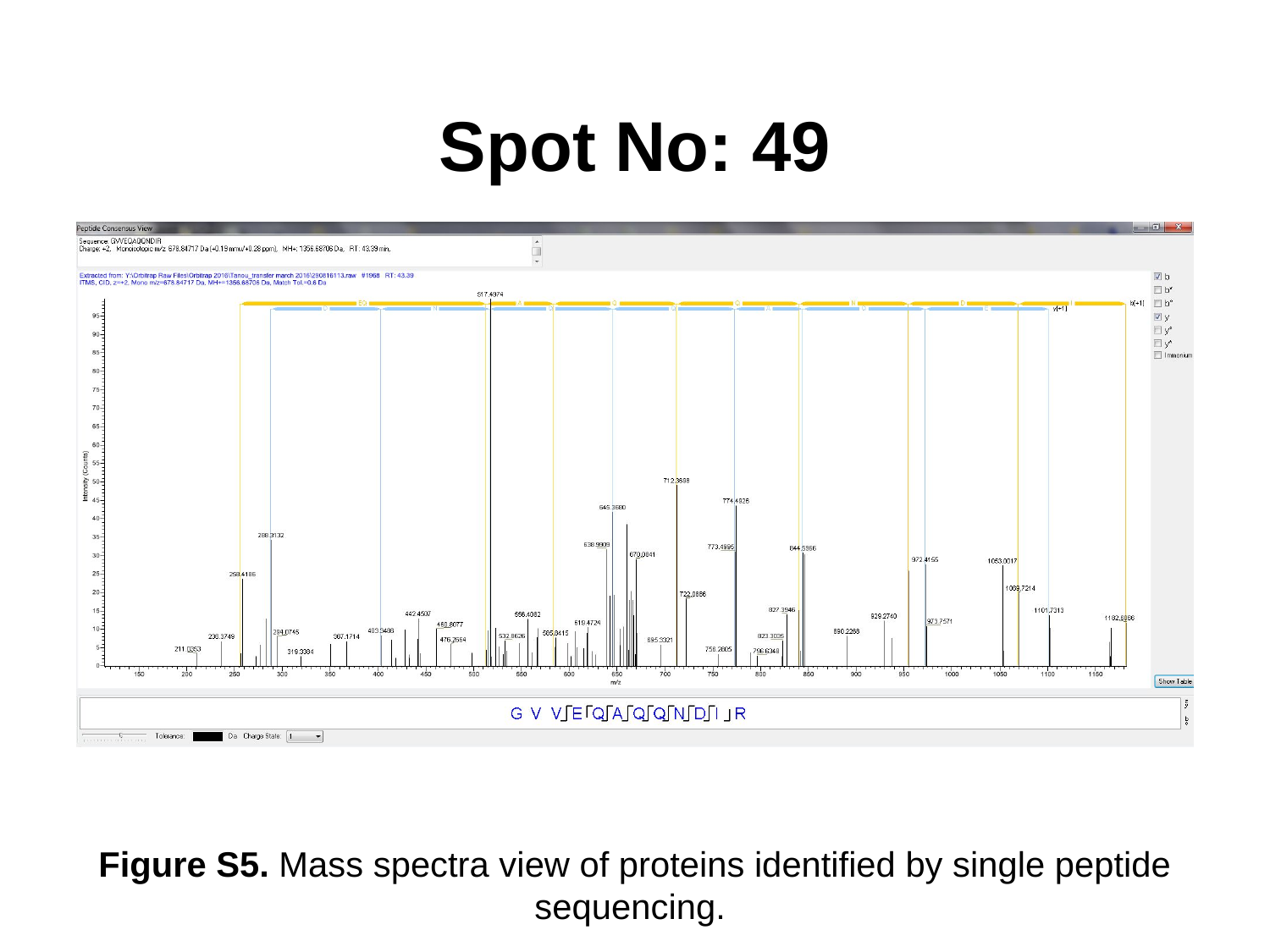

# Spot No: 49
Figure S5. Mass spectra view of proteins identified by single peptide sequencing.

## Slide 2
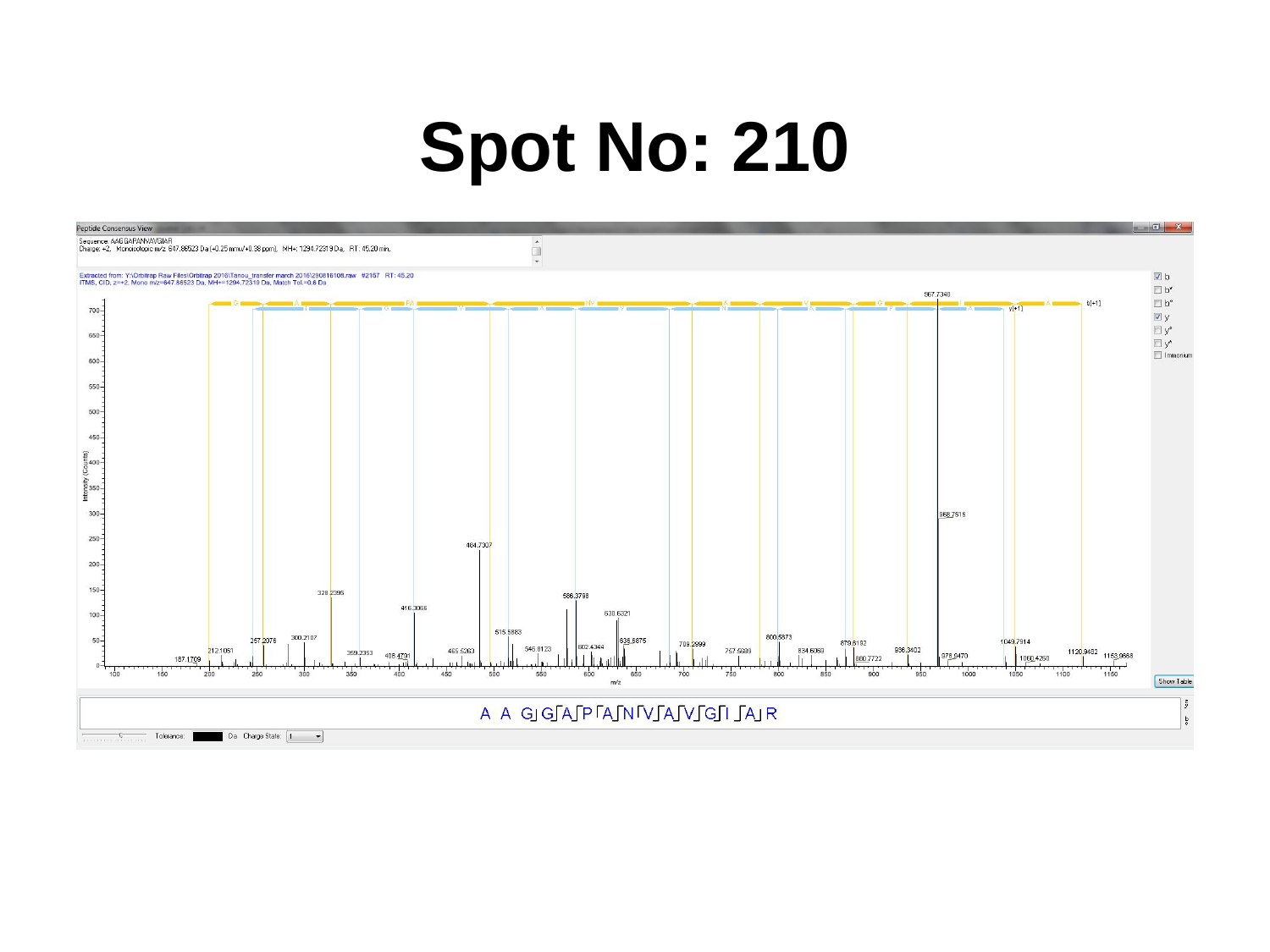

# Spot No: 210

## Slide 3
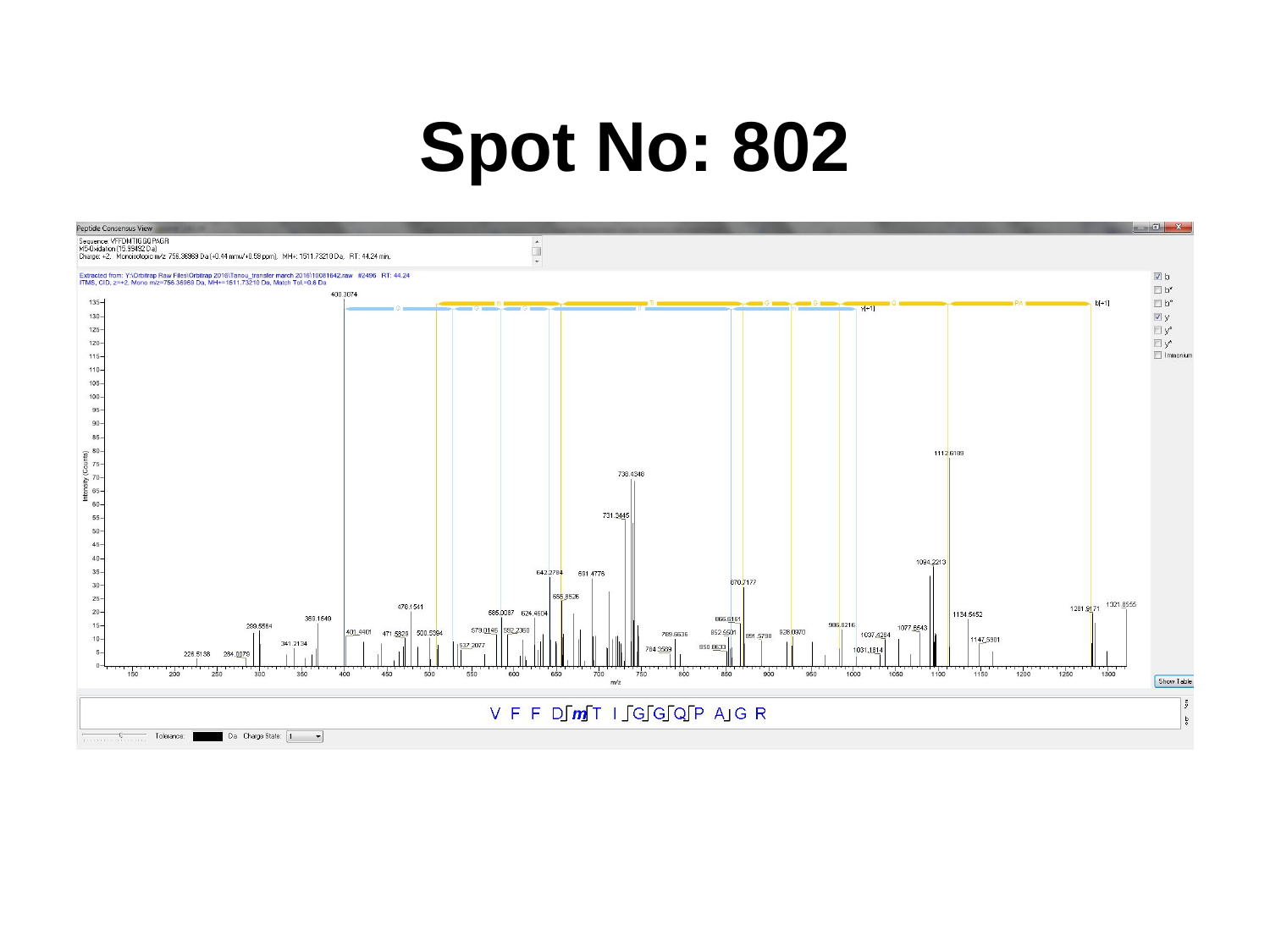

# Spot No: 802

## Slide 4
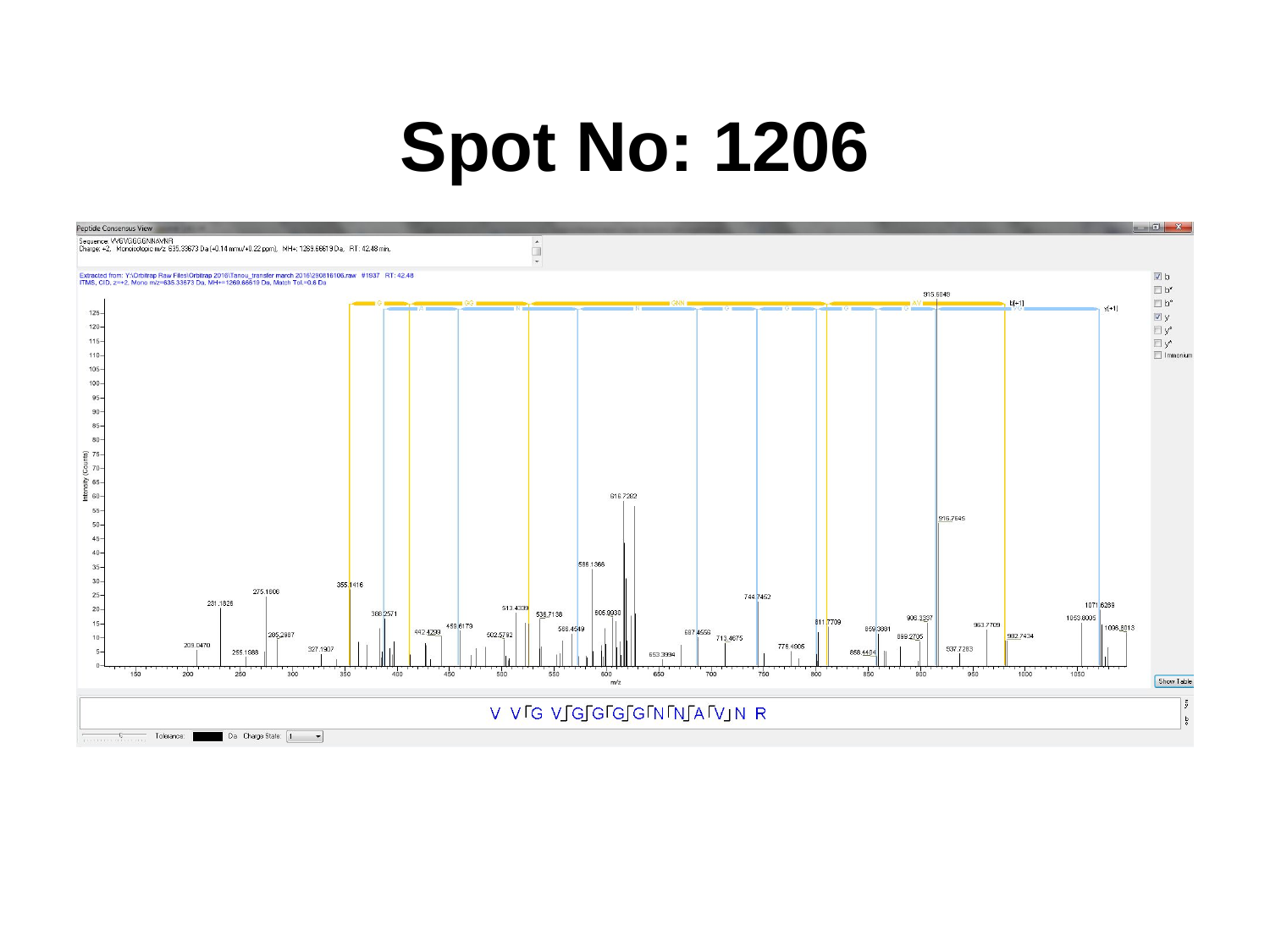

# Spot No: 1206

## Slide 5
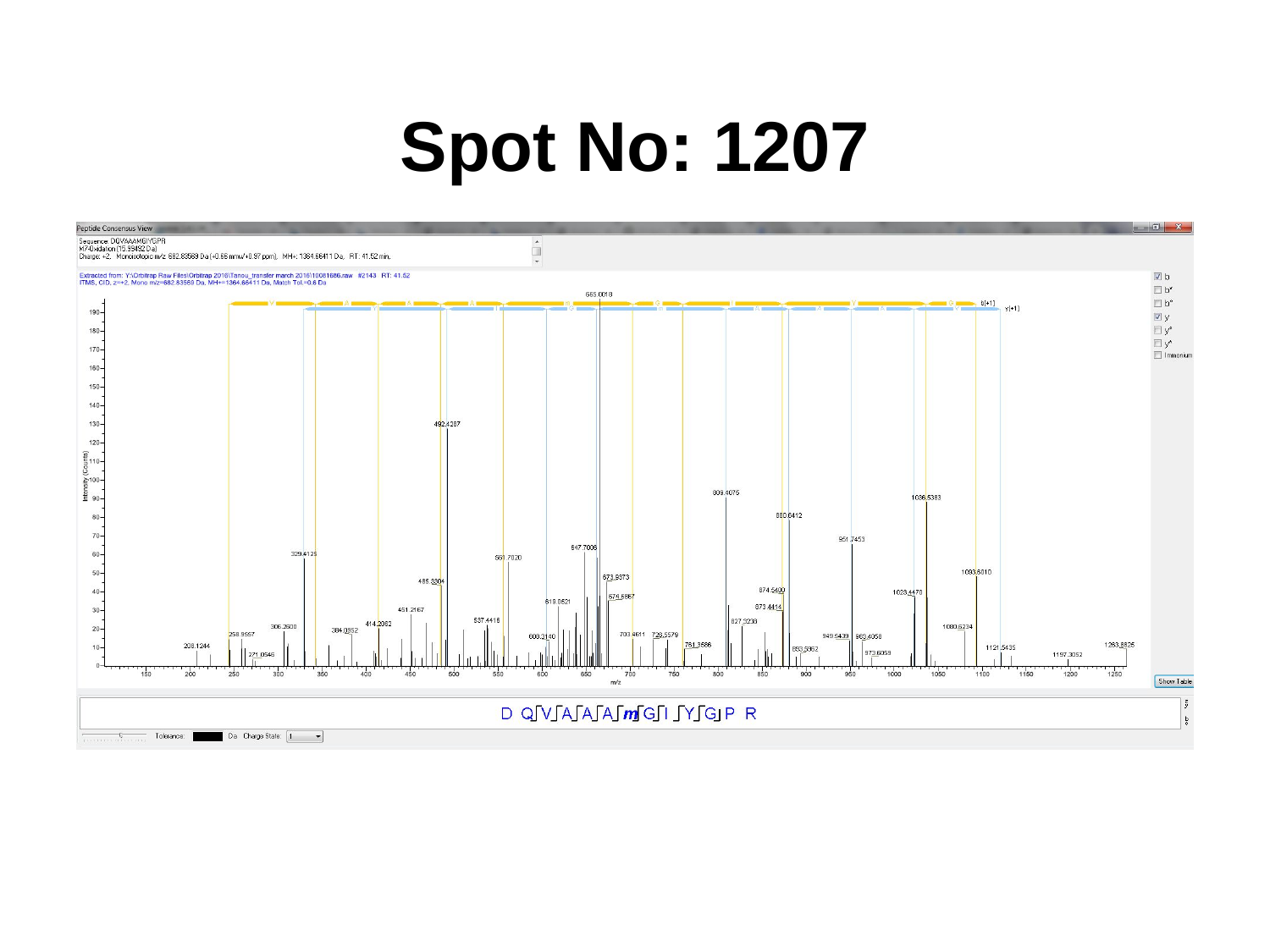

# Spot No: 1207

## Slide 6
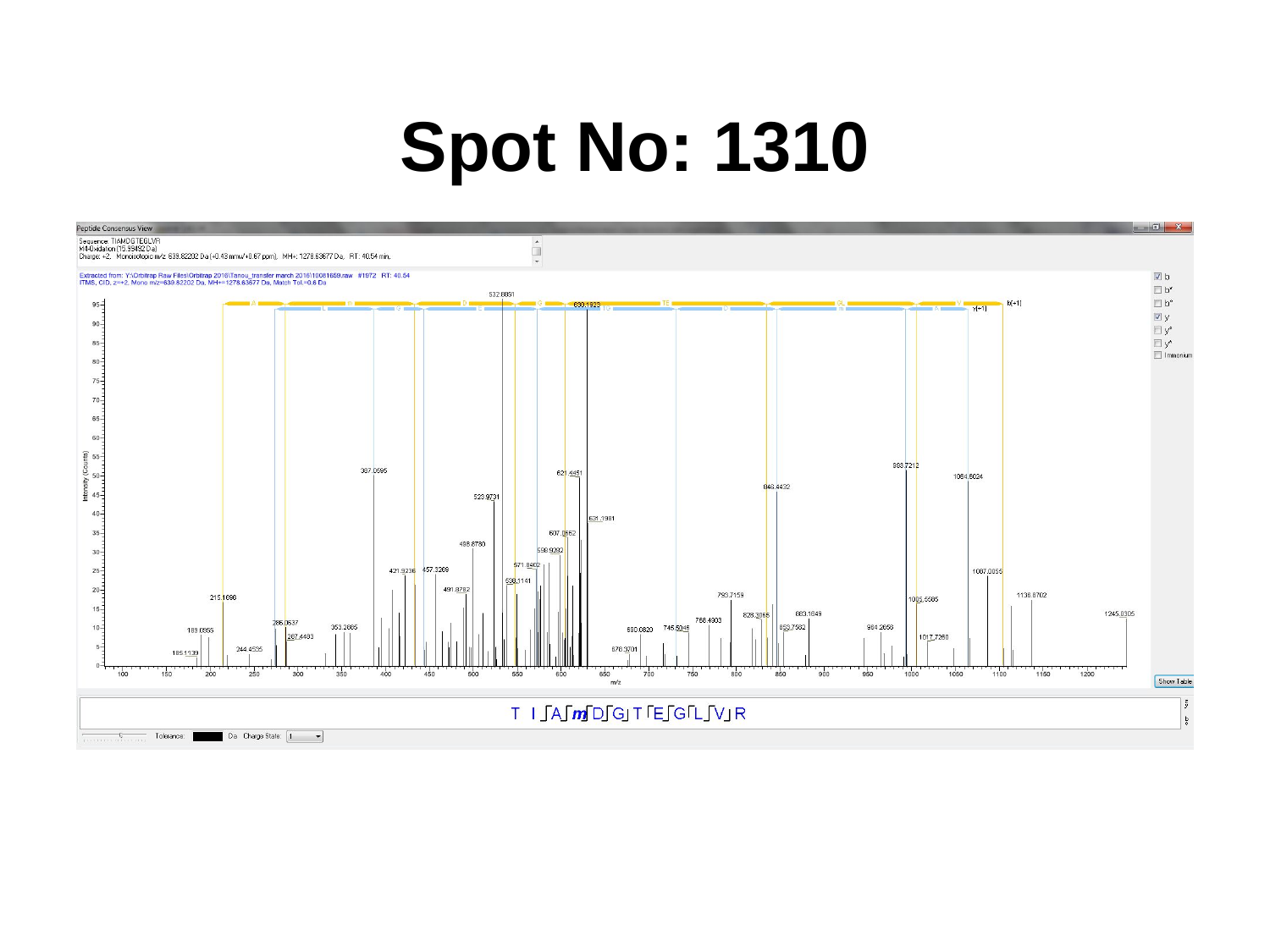

# Spot No: 1310

## Slide 7
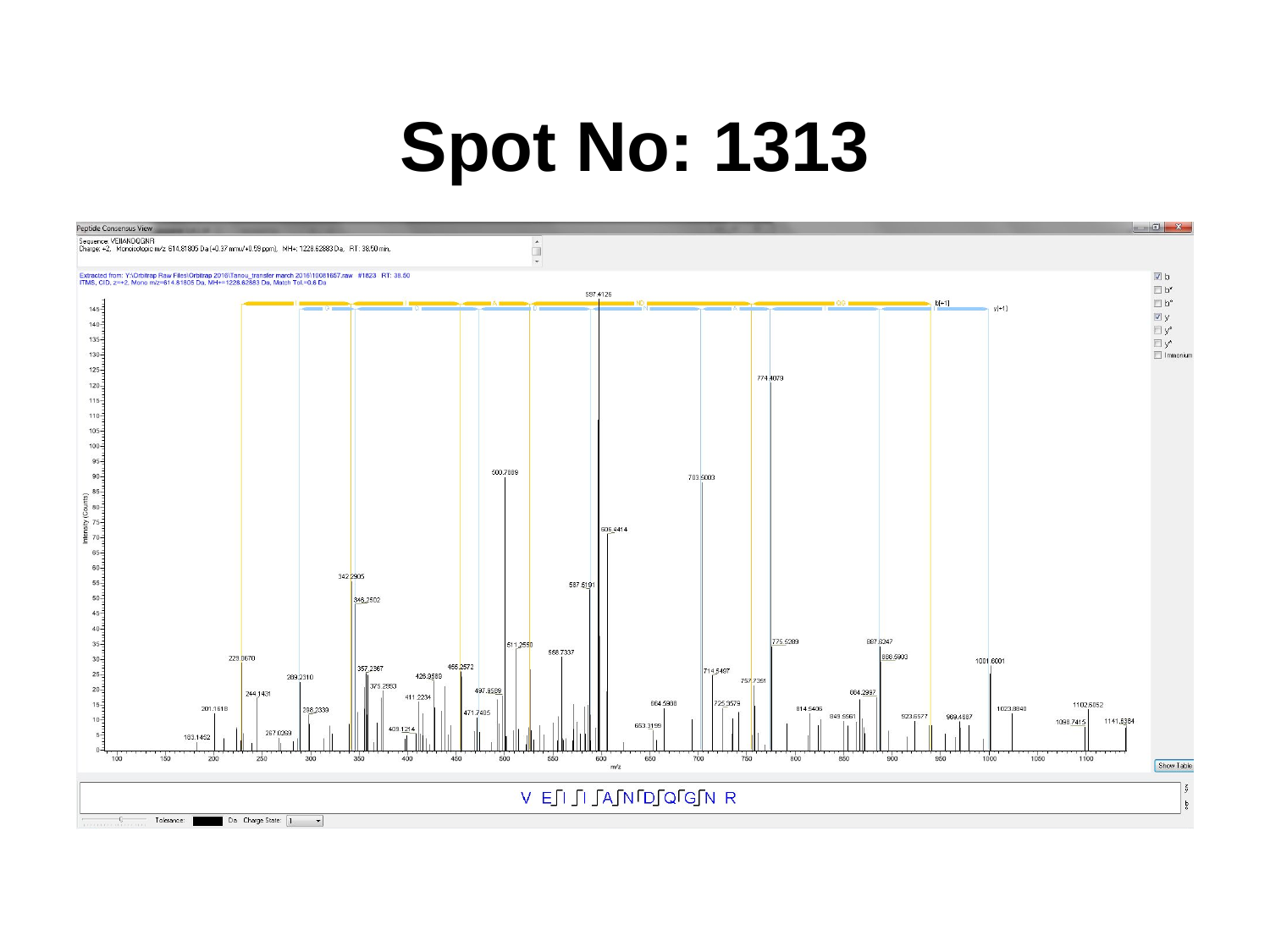

# Spot No: 1313

## Slide 8
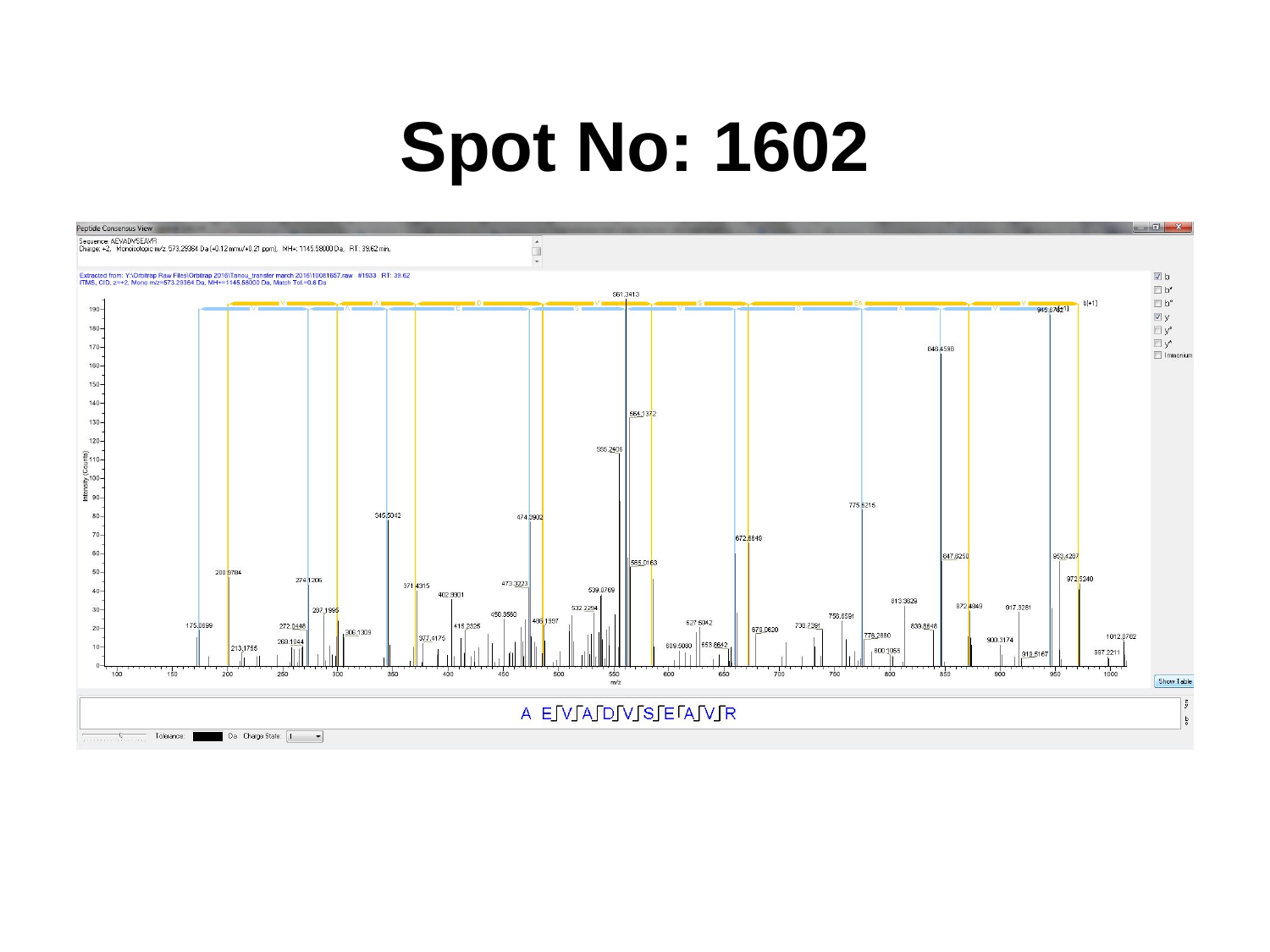

# Spot No: 1602

## Slide 9
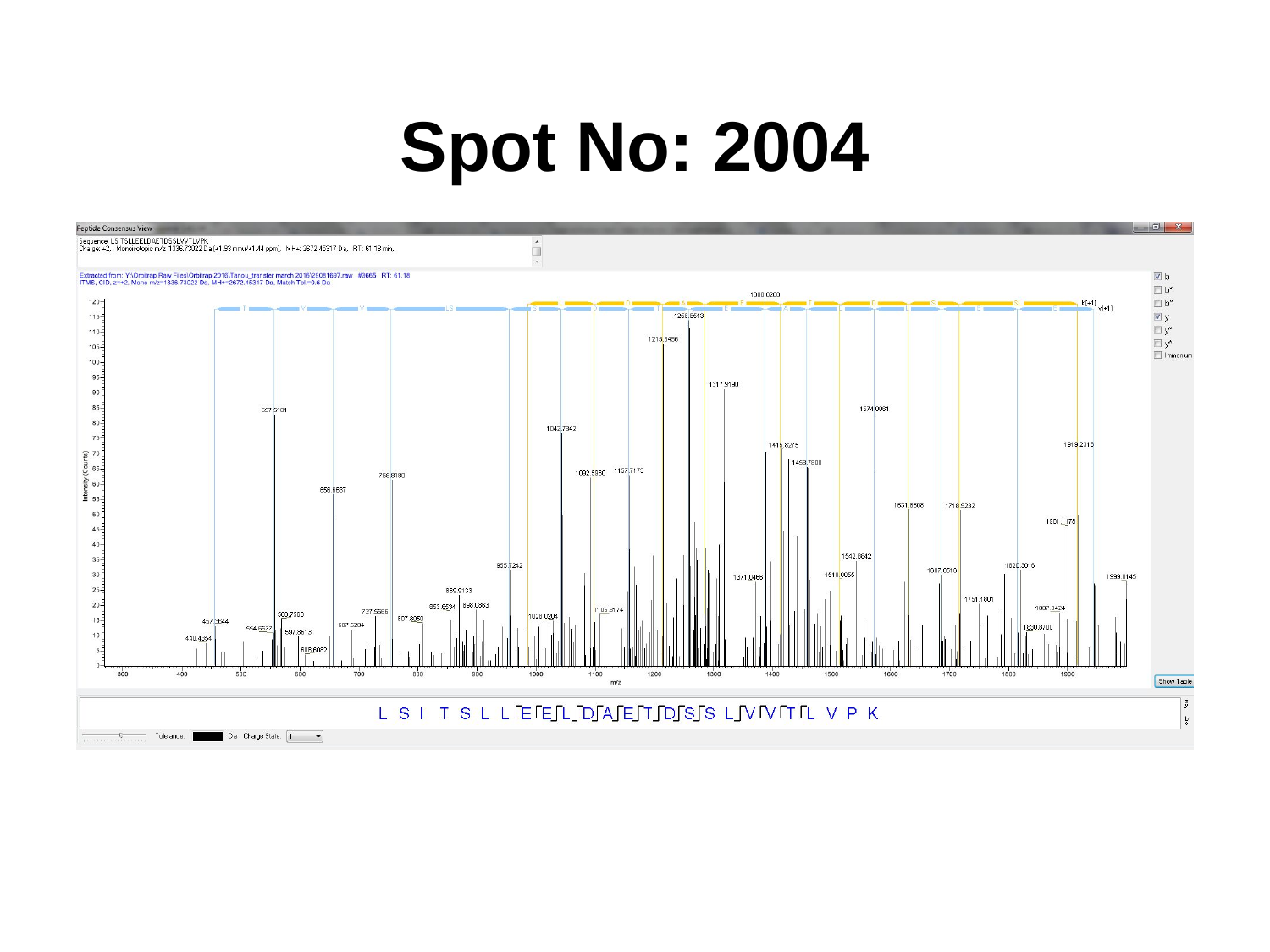

# Spot No: 2004

## Slide 10
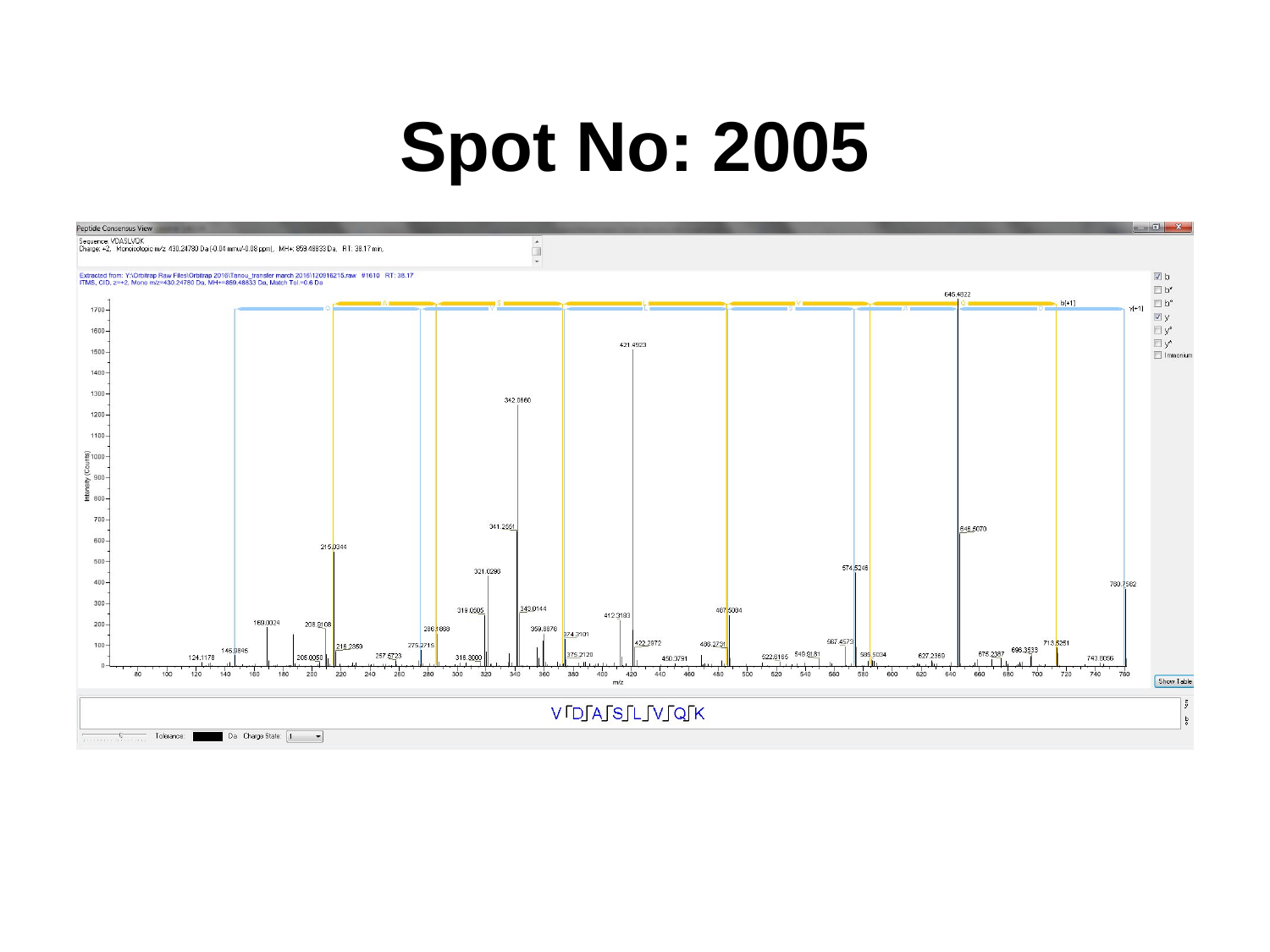

Spot No: 2005

## Slide 11
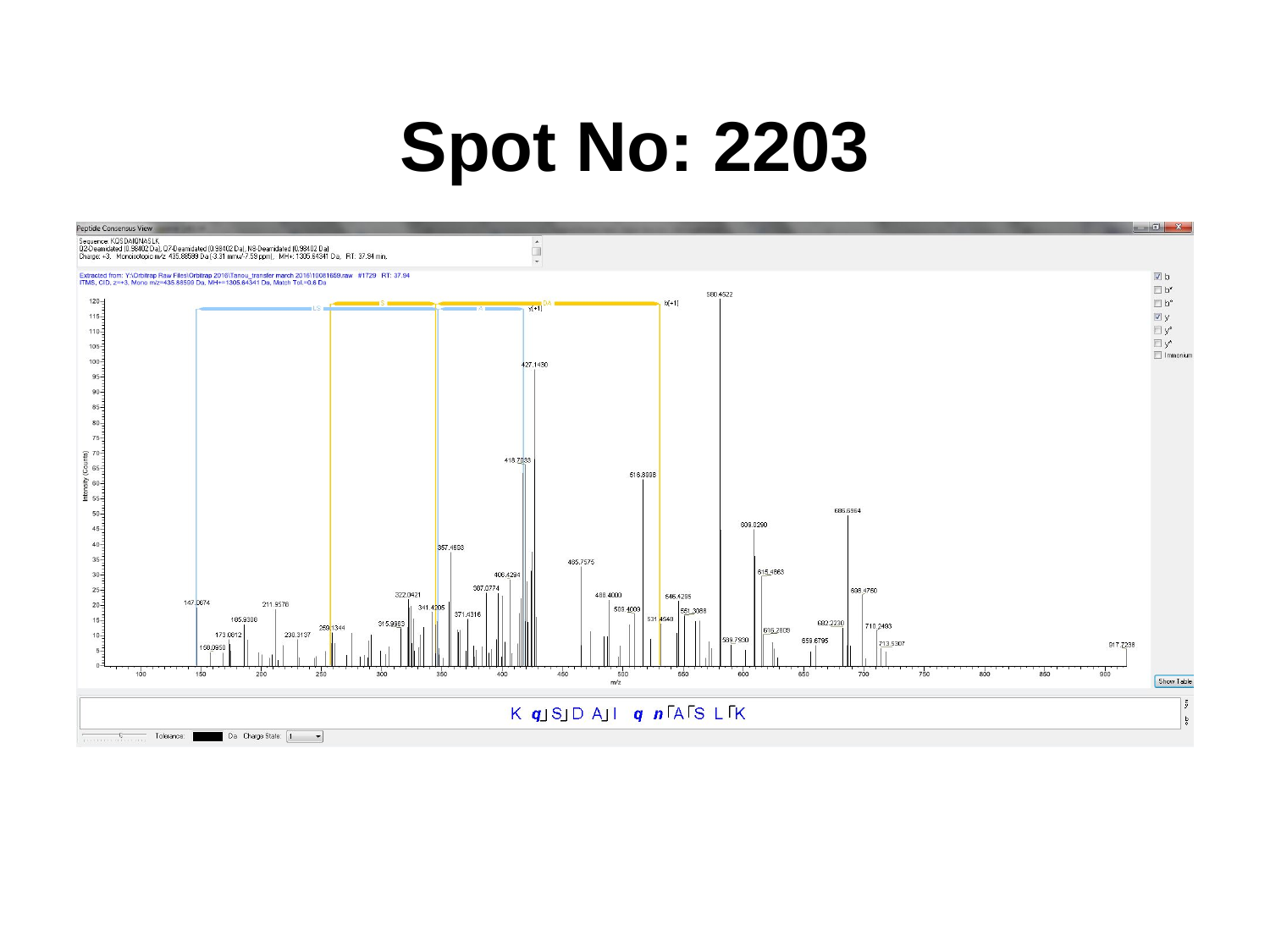

# Spot No: 2203

## Slide 12
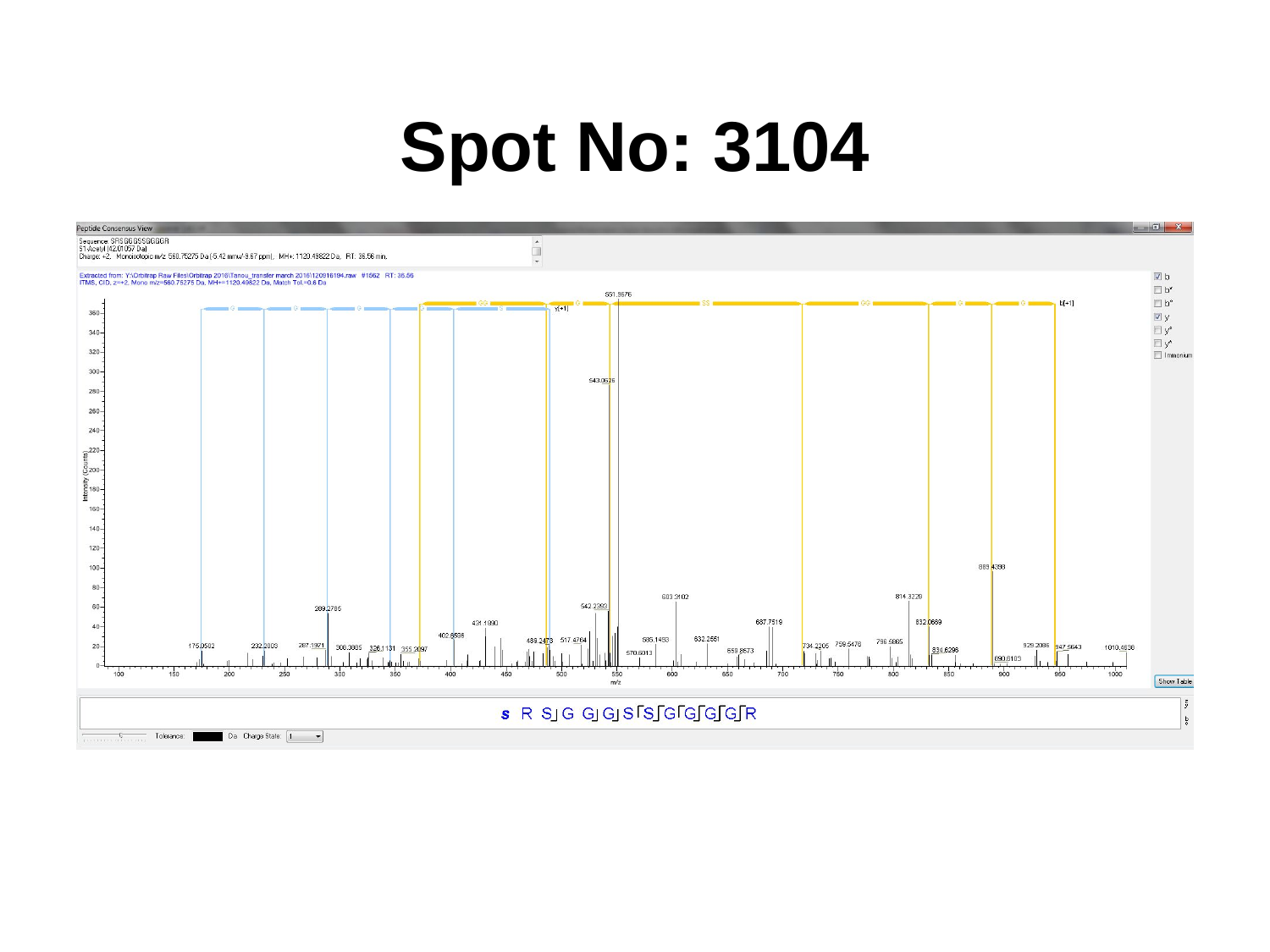

# Spot No: 3104

## Slide 13
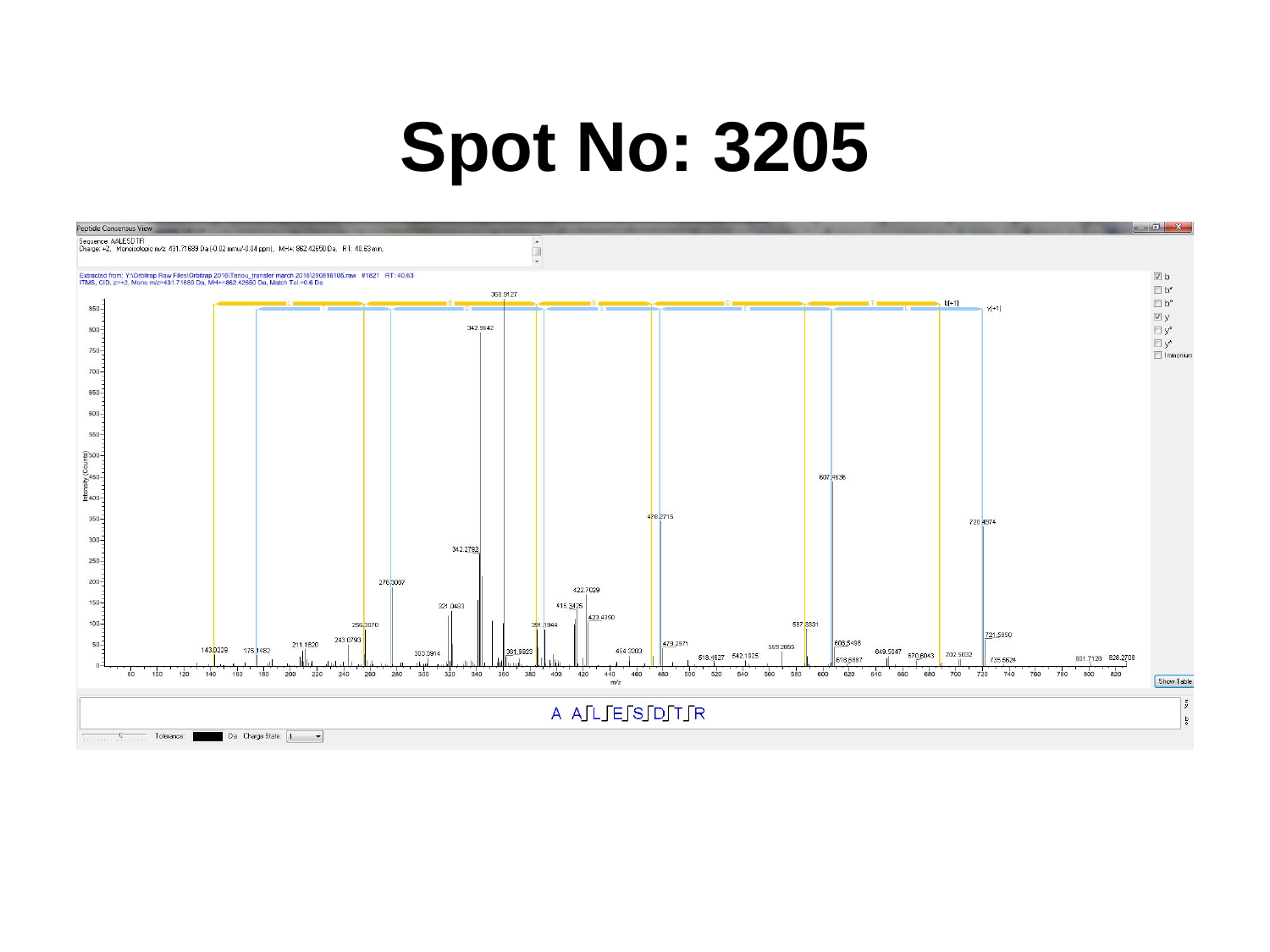

# Spot No: 3205

## Slide 14
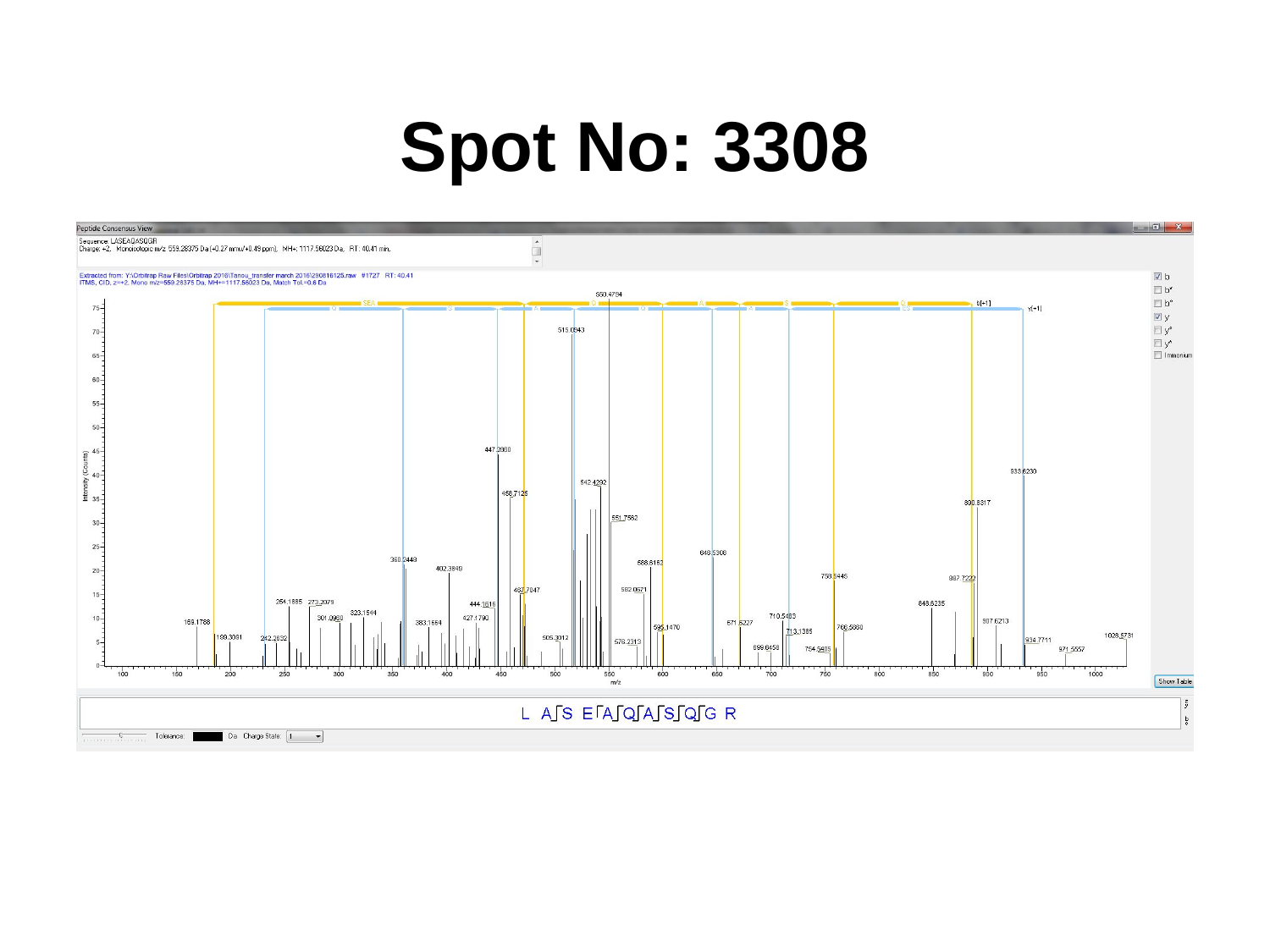

# Spot No: 3308

## Slide 15
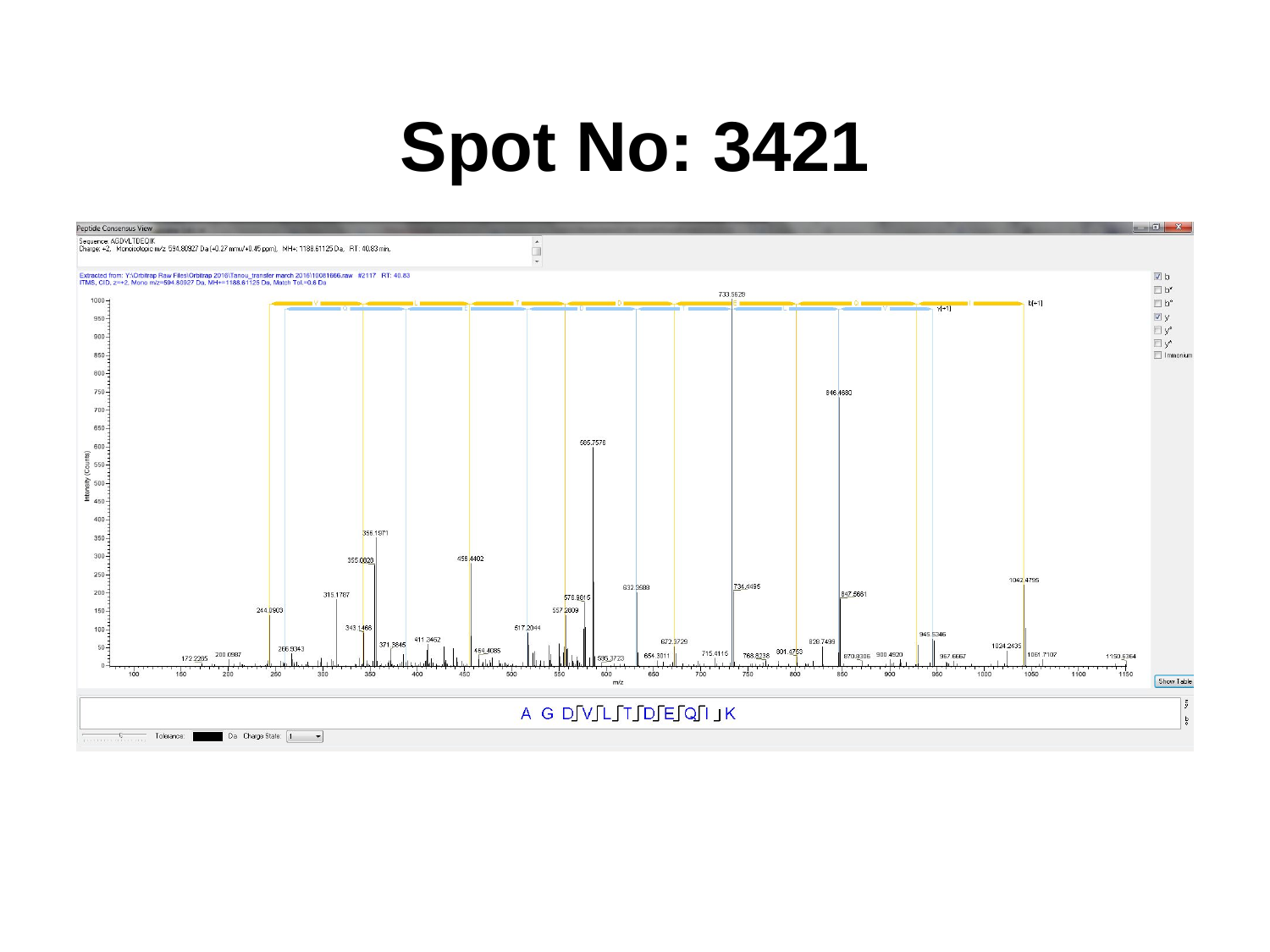

# Spot No: 3421

## Slide 16
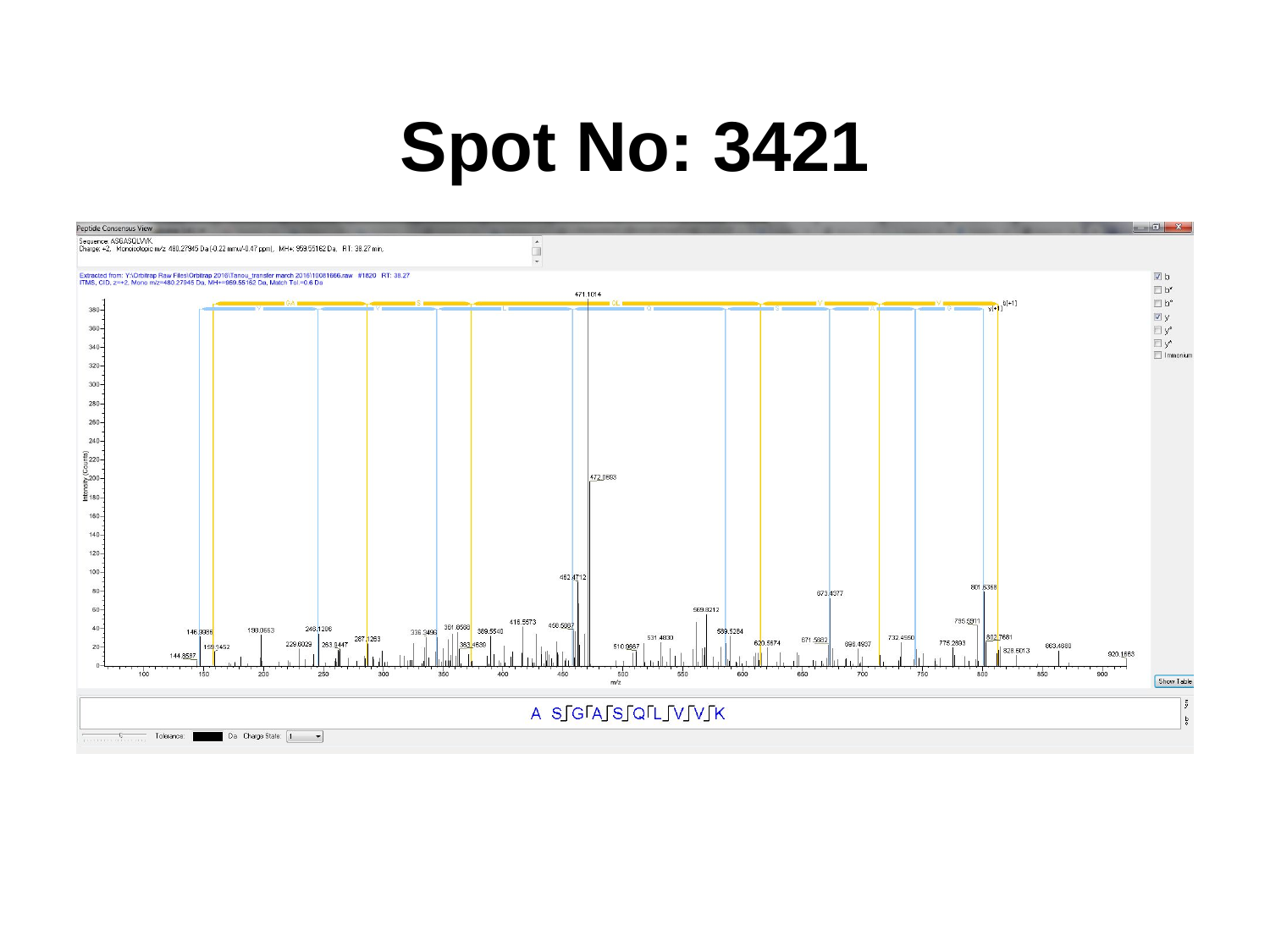

# Spot No: 3421

## Slide 17
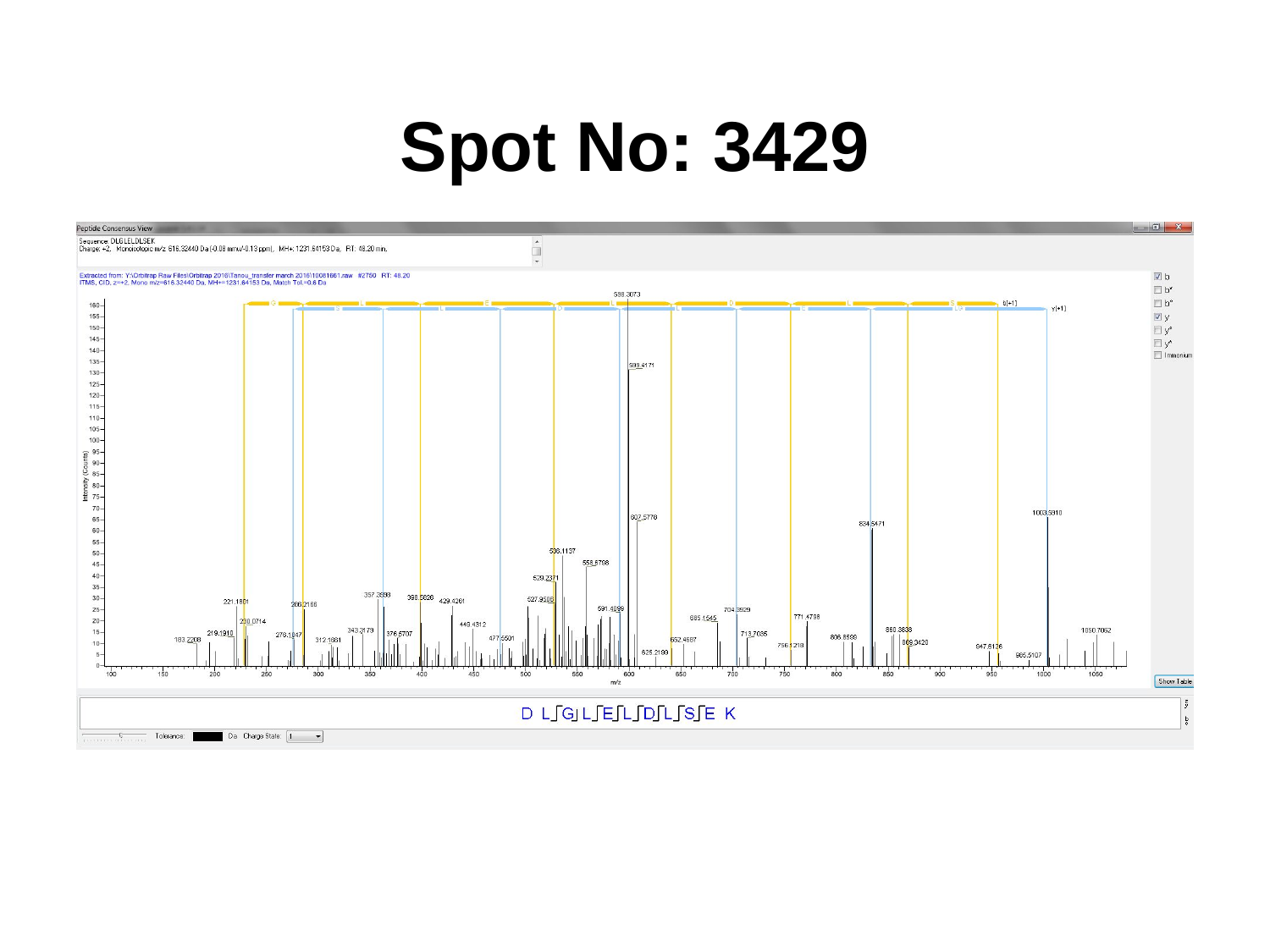

# Spot No: 3429

## Slide 18
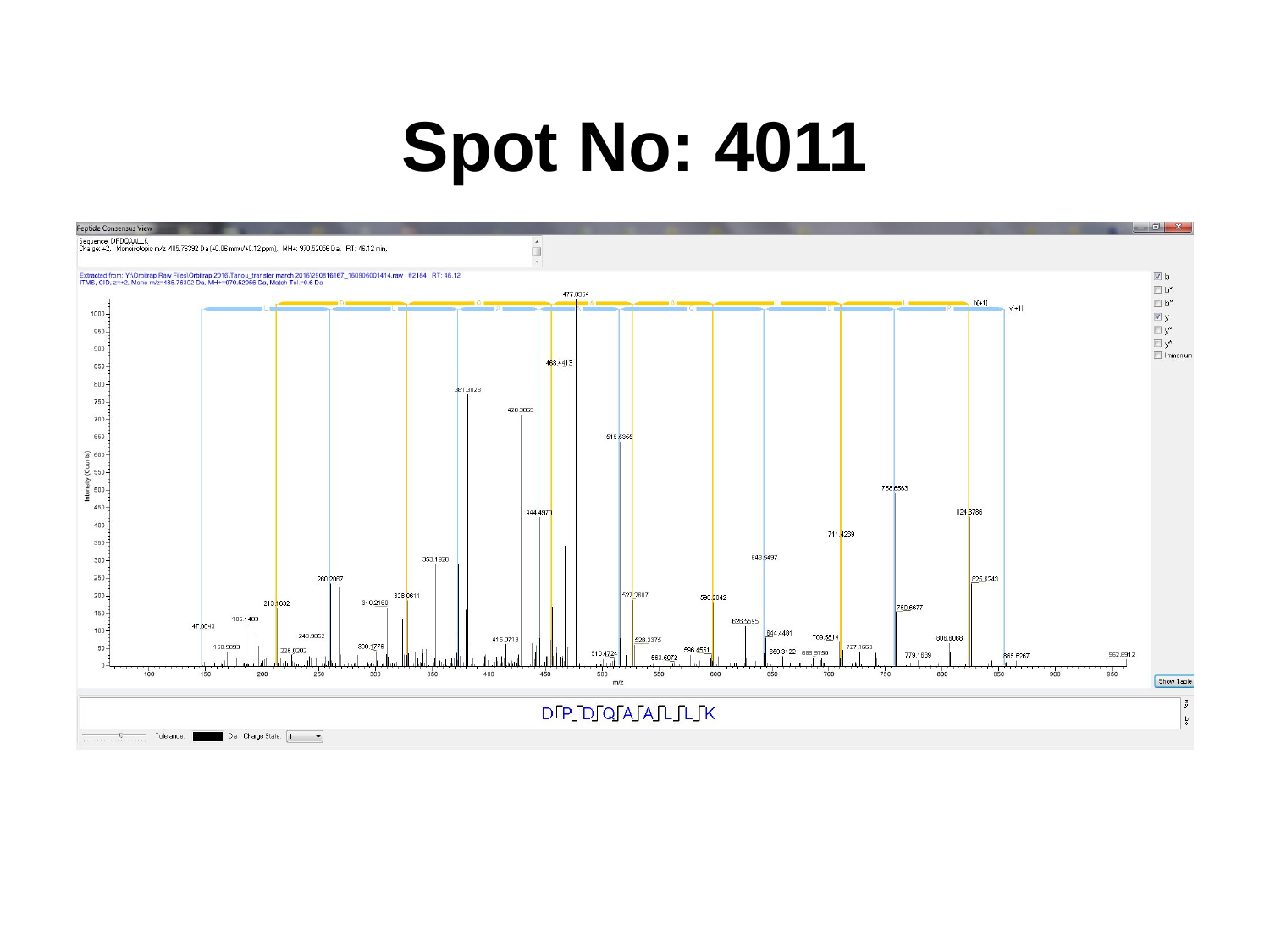

# Spot No: 4011

## Slide 19
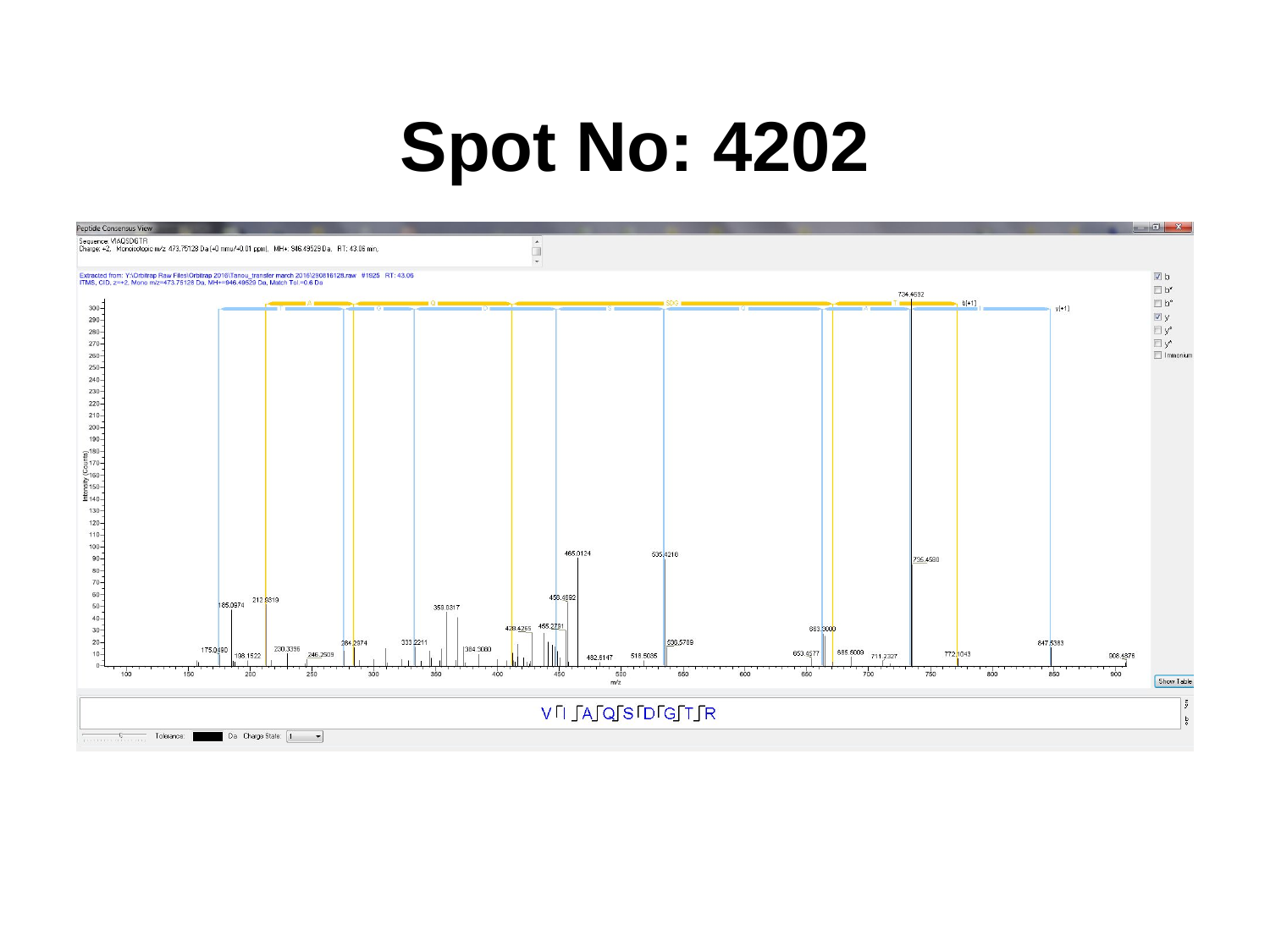

# Spot No: 4202

## Slide 20
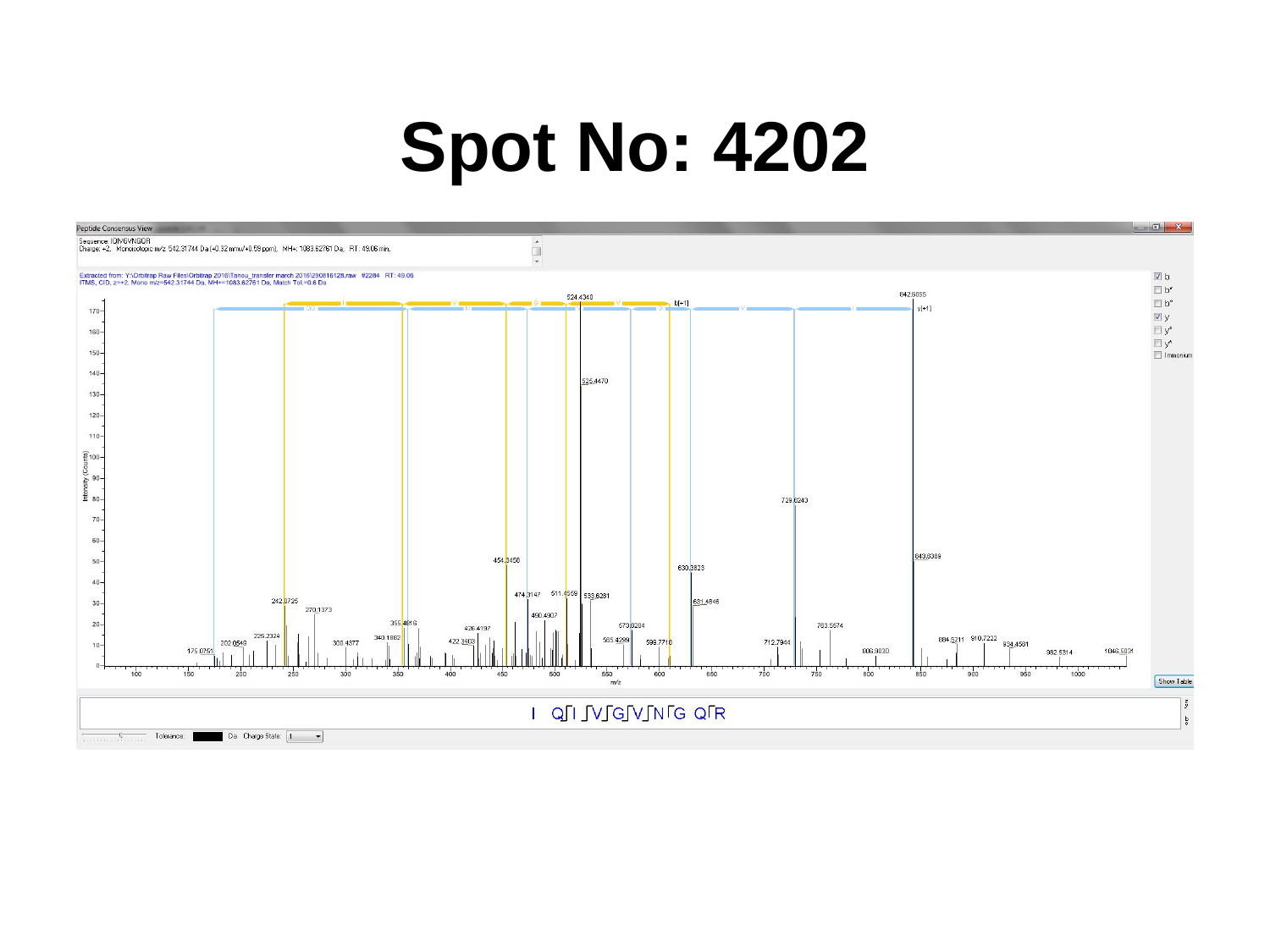

# Spot No: 4202

## Slide 21
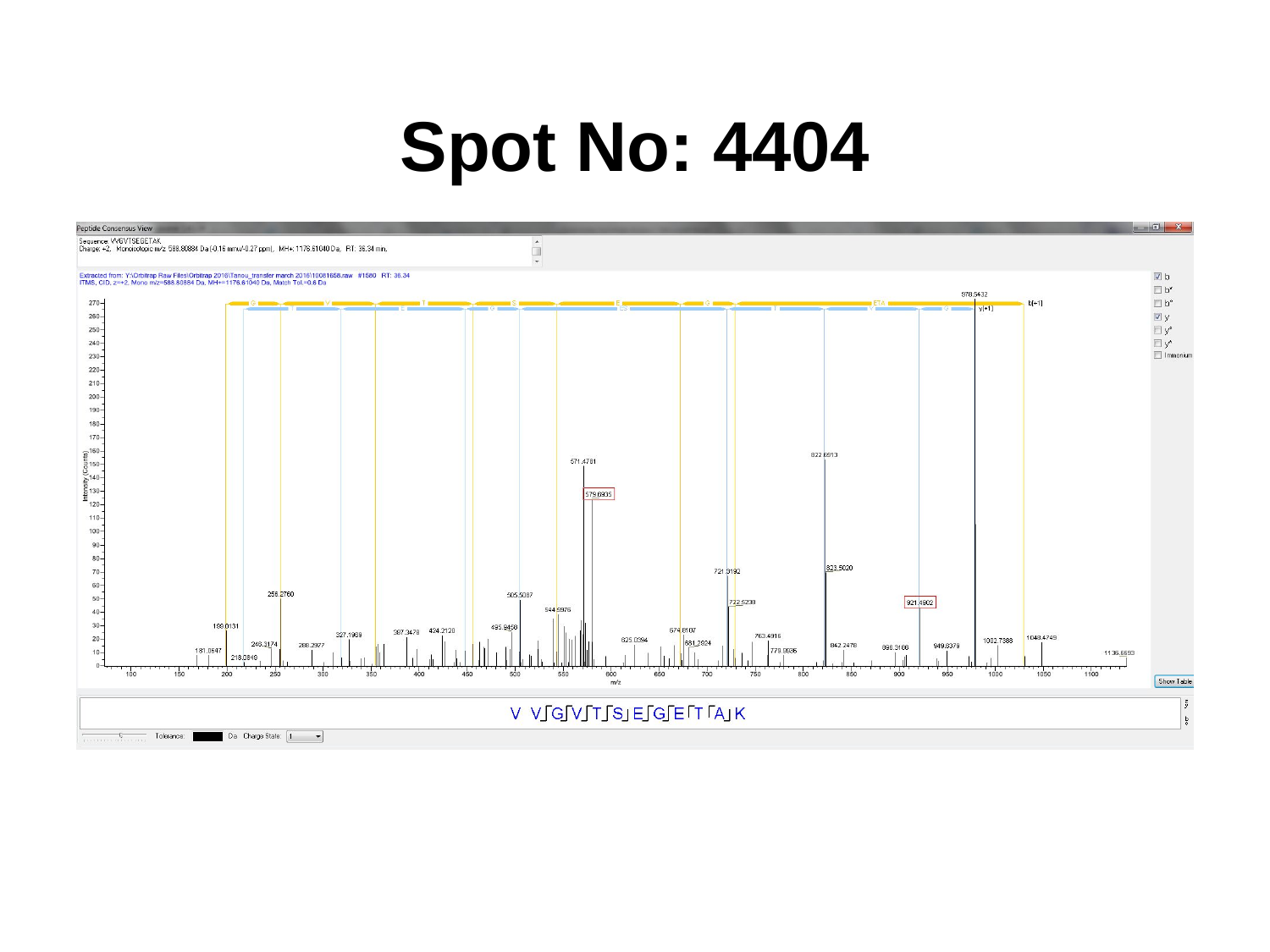

# Spot No: 4404

## Slide 22
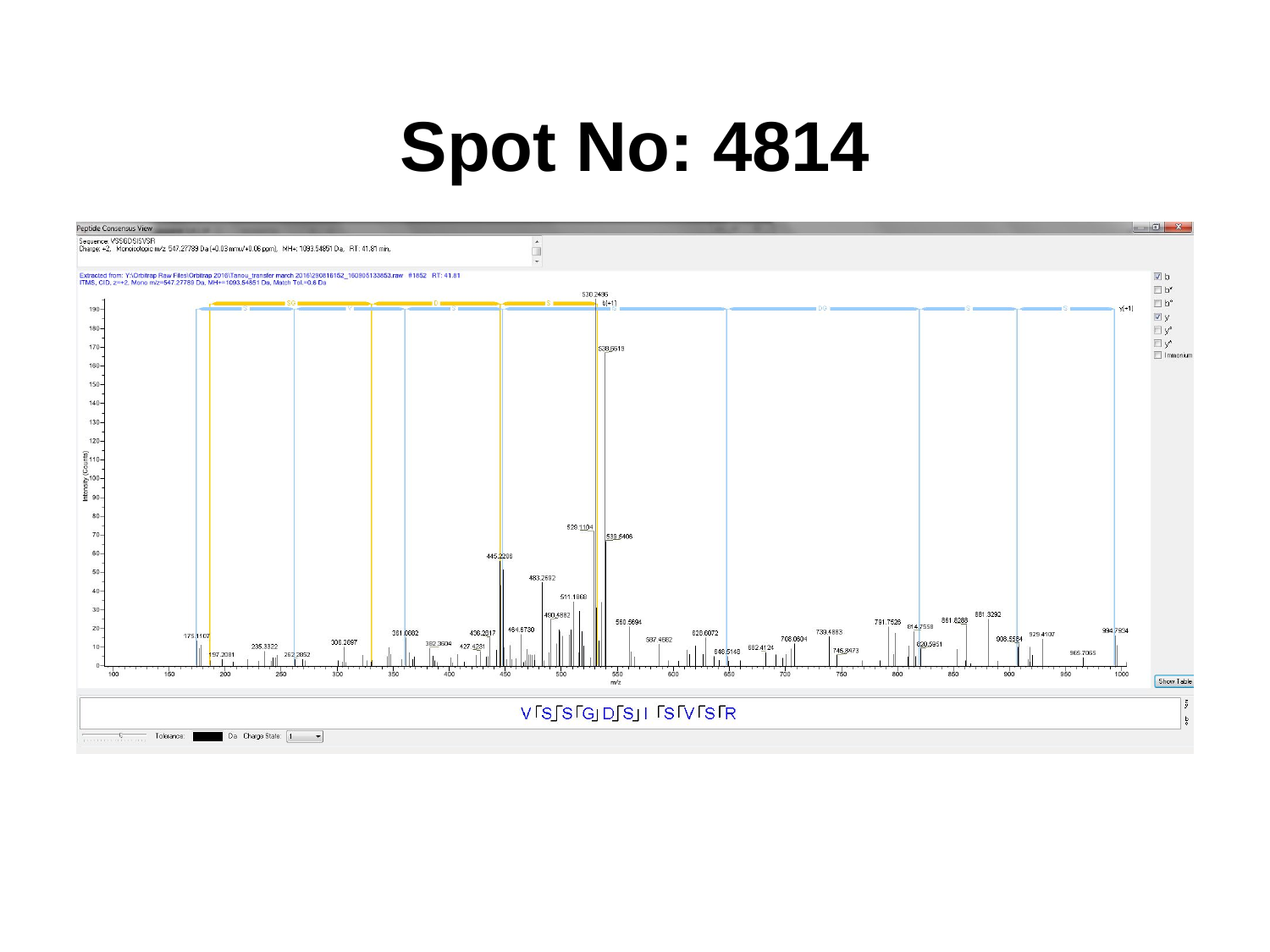

# Spot No: 4814

## Slide 23
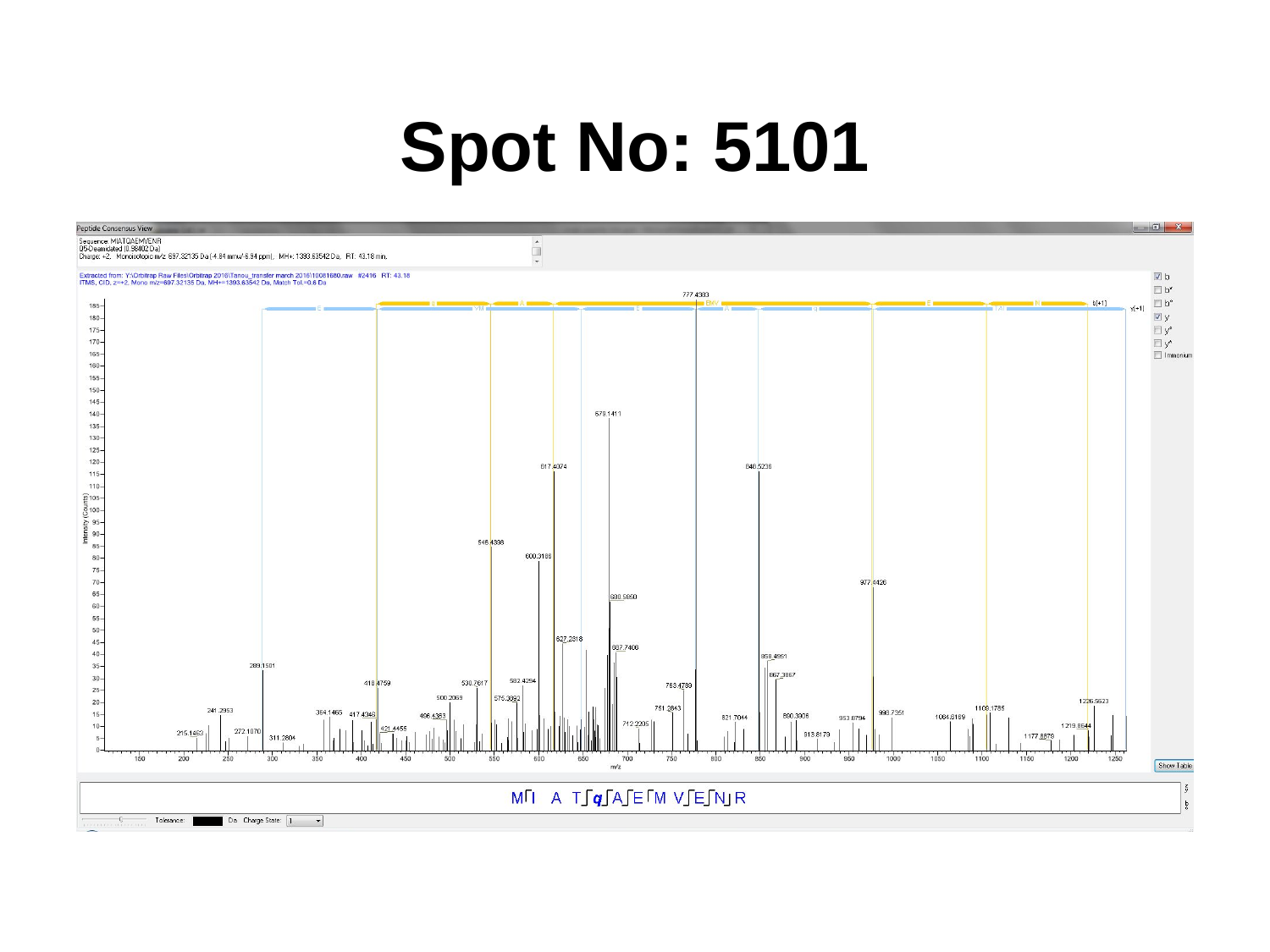

# Spot No: 5101

## Slide 24
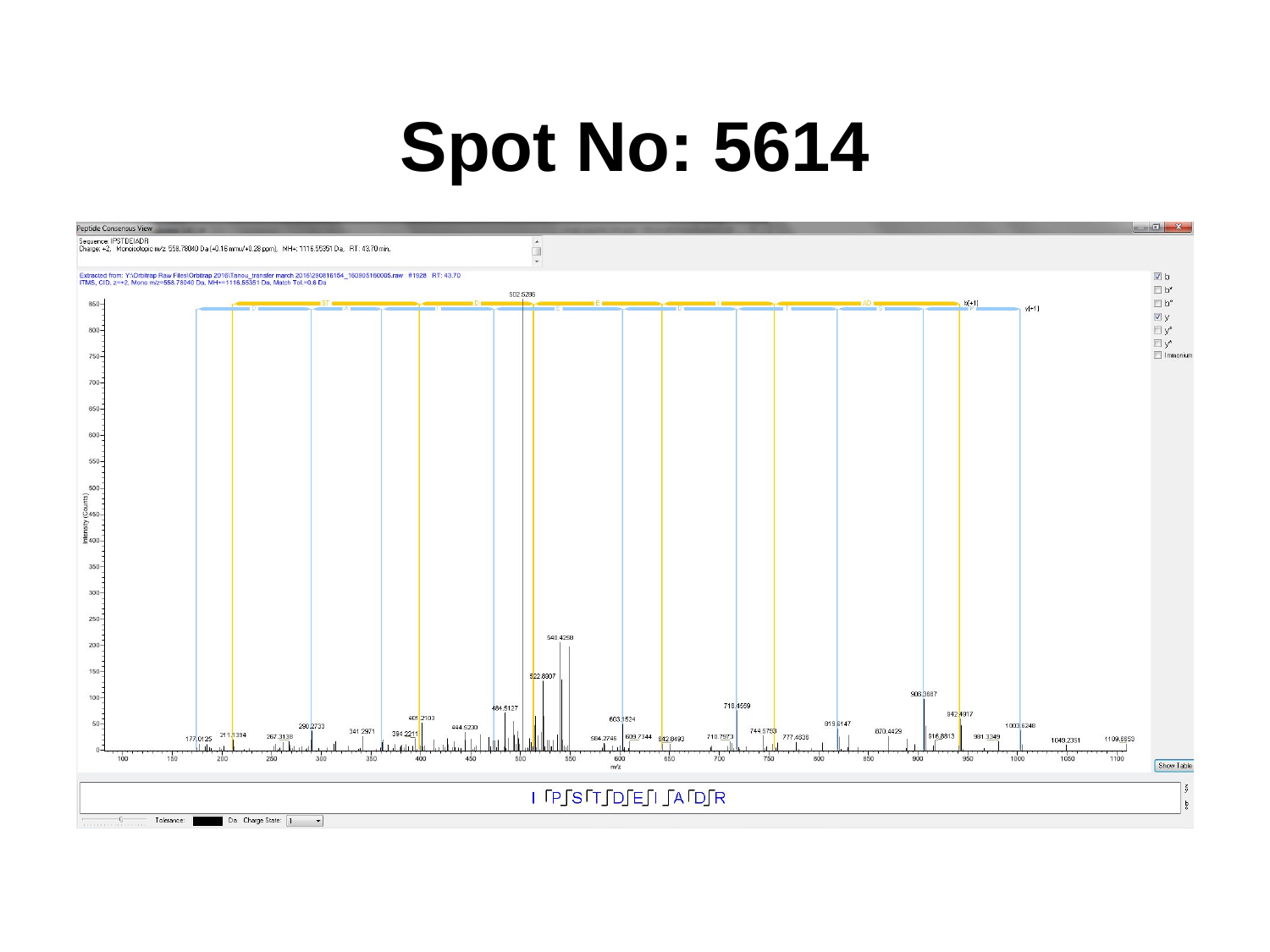

# Spot No: 5614

## Slide 25
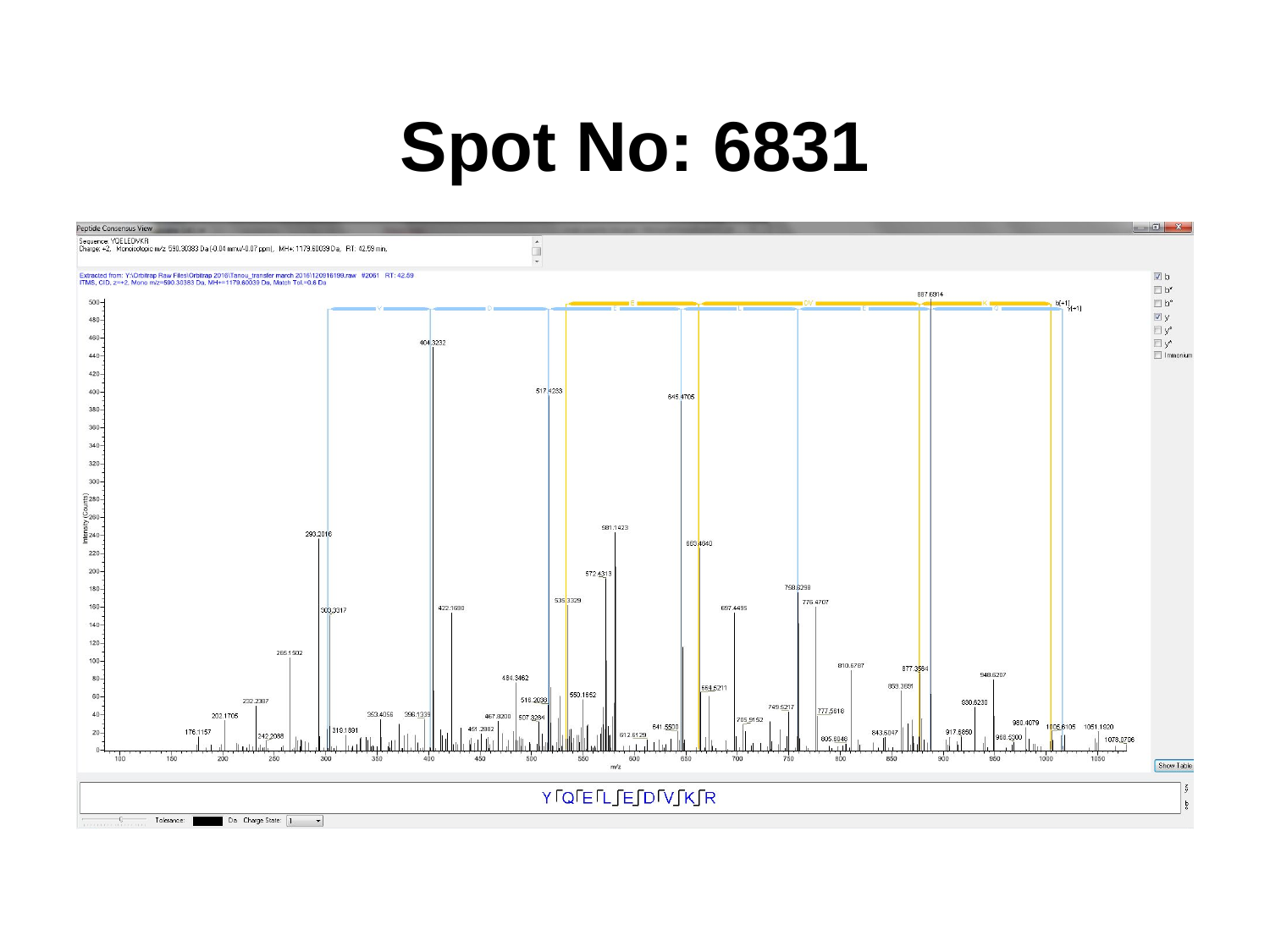

# Spot No: 6831

## Slide 26
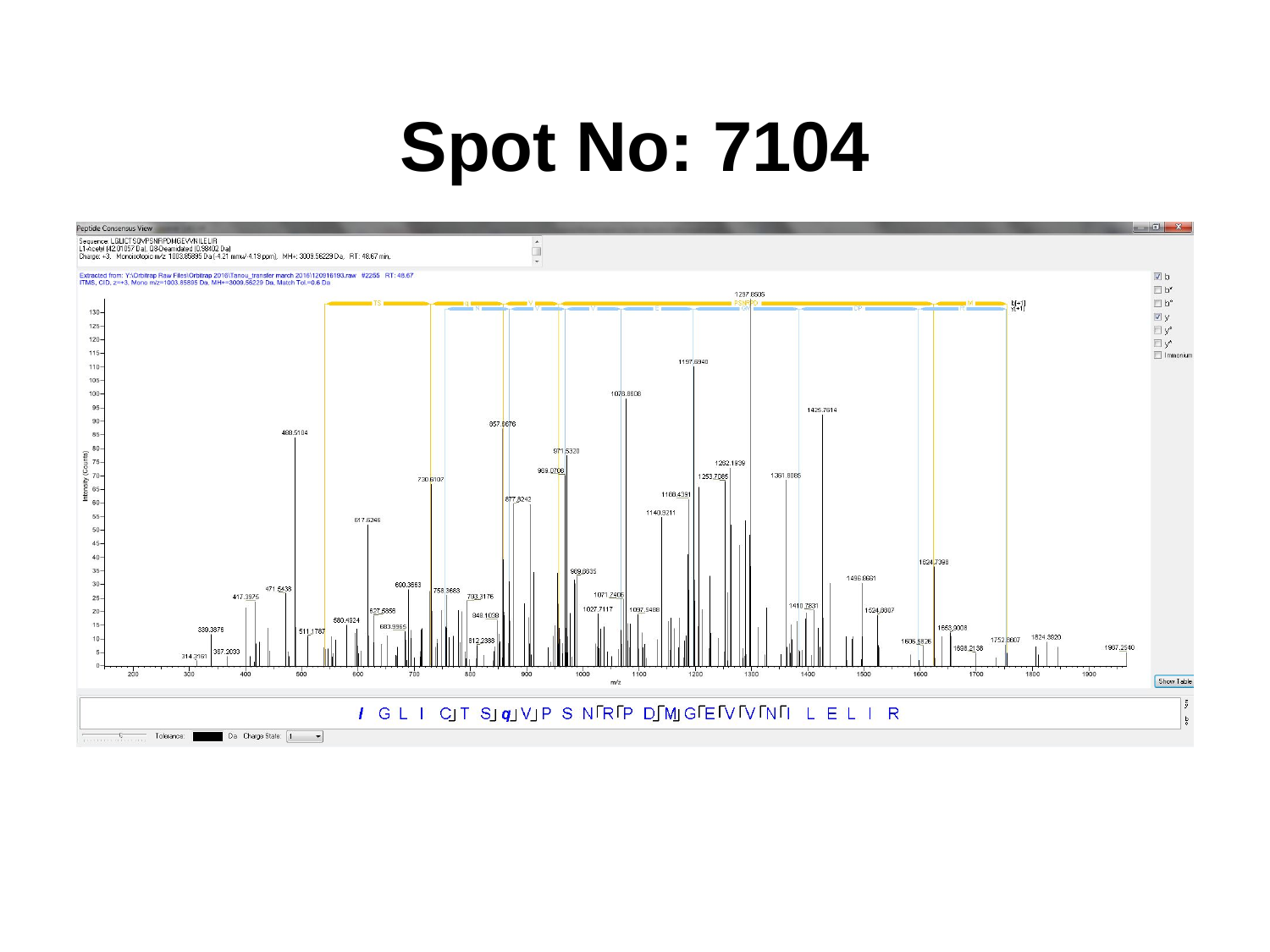

# Spot No: 7104

## Slide 27
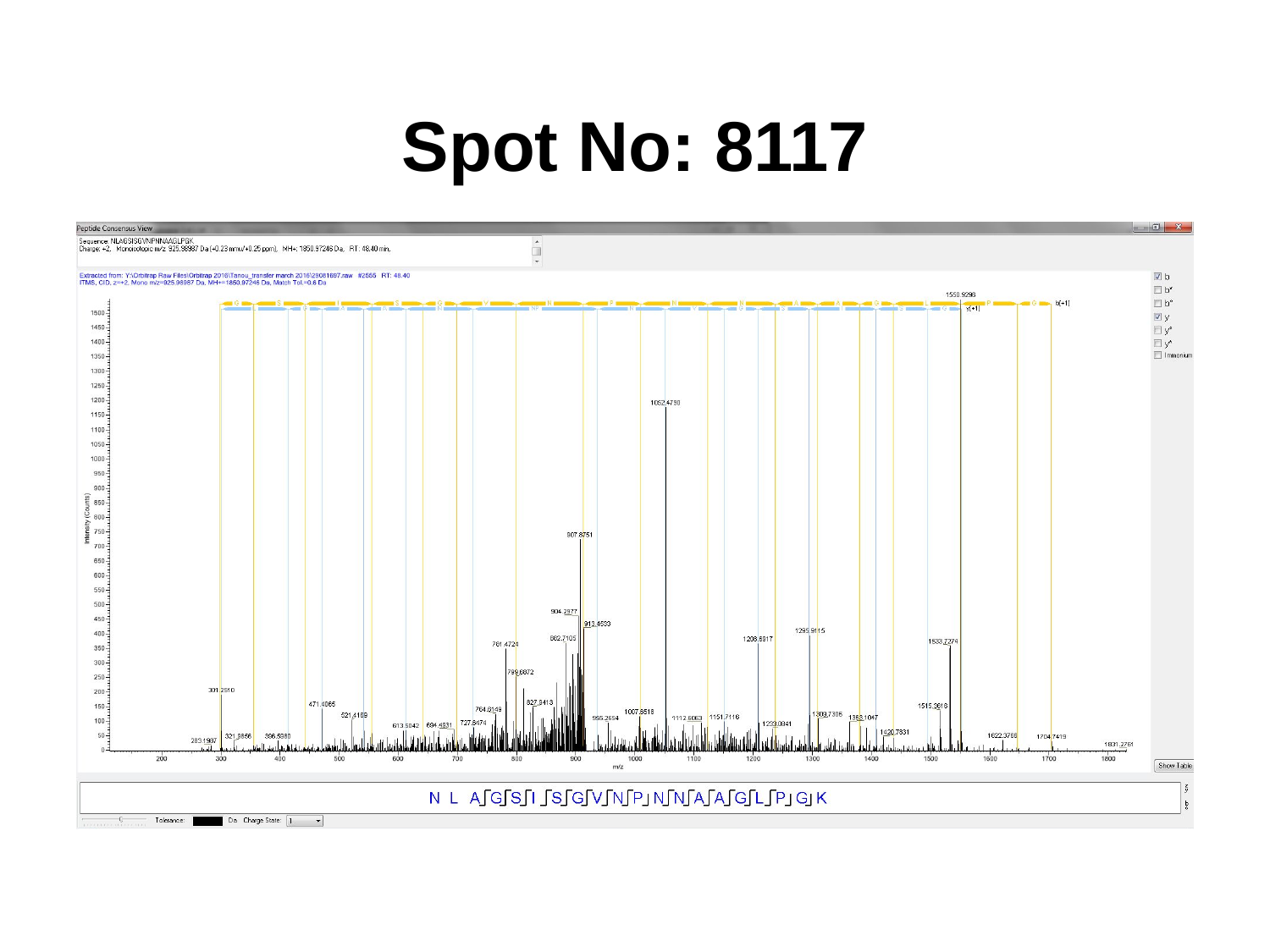

# Spot No: 8117

## Slide 28
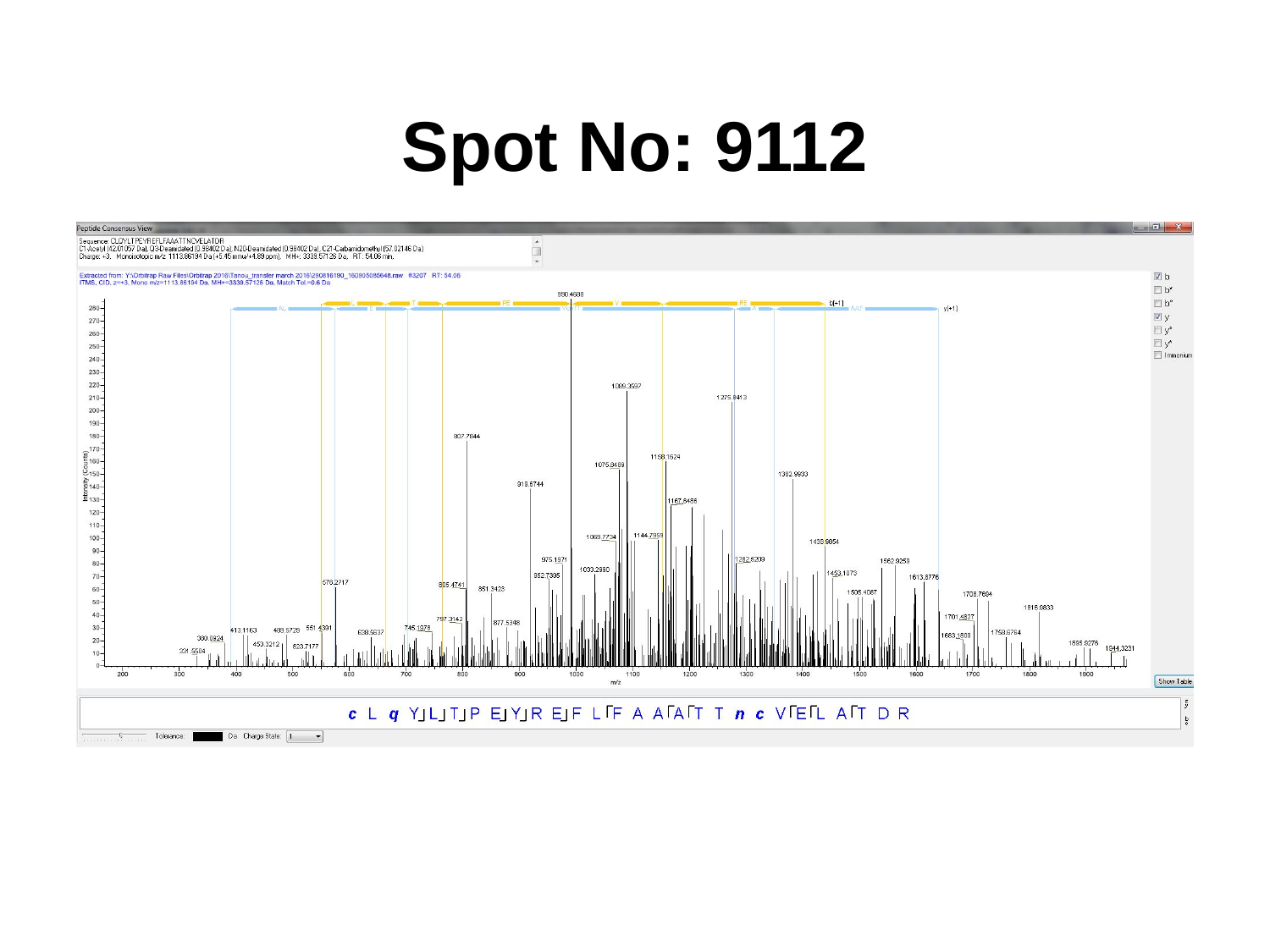

# Spot No: 9112

## Slide 29
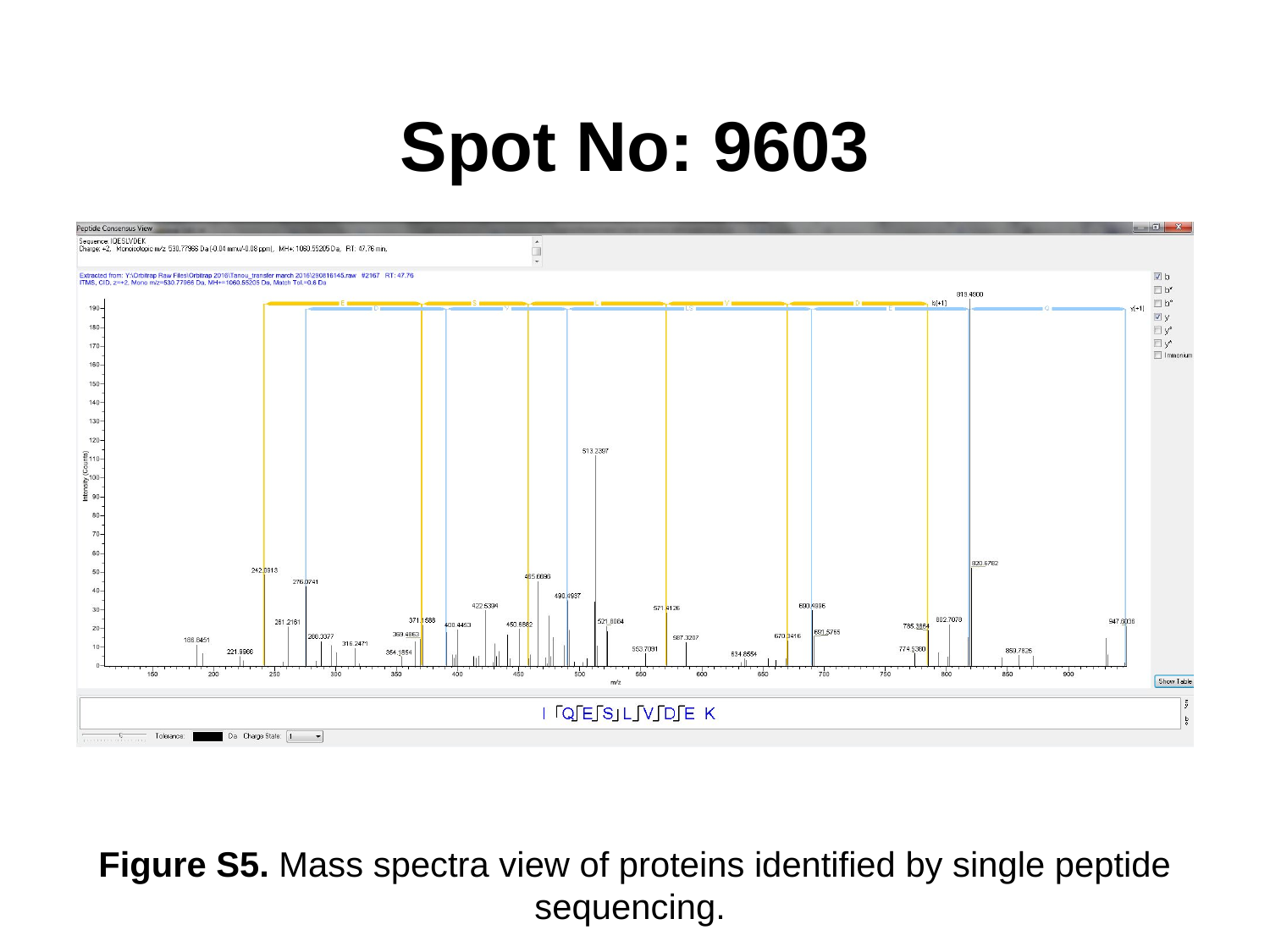

# Spot No: 9603
Figure S5. Mass spectra view of proteins identified by single peptide sequencing.
